# The Wilms Tumor Gene, *Wt1*, Is Critical for Mouse Spermatogenesis via Regulation of Sertoli Cell Polarity and Is Associated with Non-Obstructive Azoospermia in Humans

**DOI:** 10.1371/journal.pgen.1003645

**Published:** 2013-08-01

**Authors:** Xiao Na Wang, Ze Song Li, Yu Ren, Tao Jiang, Ya Qing Wang, Min Chen, Jun Zhang, Jian Xiu Hao, Yan Bo Wang, Ri Na Sha, Yi Huang, Xiao Liu, Jing Chu Hu, Guang Qing Sun, Hong Gang Li, Cheng Liang Xiong, Jun Xie, Zhi Mao Jiang, Zhi Ming Cai, Jun Wang, Jian Wang, Vicki Huff, Yao Ting Gui, Fei Gao

**Affiliations:** 1 State Key Laboratory of Reproductive Biology, Institute of Zoology, Chinese Academy of Sciences, Beijing, China; 2 University of Chinese Academy of Sciences, Beijing, China; 3 Guangdong and Shenzhen Key Laboratory of Male Reproductive Medicine and Genetics, Institute of Urology, Peking University Shenzhen Hospital, Shenzhen PKU-HKUST Medical Center, Shenzhen, China; 4 Shenzhen Key Laboratory of Genitourinary Tumor, Shenzhen Second People's Hospital, the First Affiliated Hospital of Shenzhen University, Shenzhen, China; 5 Beijing Genomics Institute at Shenzhen, Shenzhen, China; 6 Key Laboratory of Molecular and Developmental Biology, Institute of Genetics and Developmental Biology, Chinese Academy of Sciences, Beijing, China; 7 Shenzhen Second People's Hospital, the First Affiliated Hospital of Shenzhen University, Shenzhen, China; 8 Center of Reproductive Medicine, Tongji Medical College, Huazhong University of Science and Technology, Wuhan, China; 9 Department of Genetics, The University of Texas M. D. Anderson Cancer Center; Graduate Programs in Human Molecular Genetics and Genes and Development, University of Texas, Houston, Texas, United States of America; Medical Research Council, United Kingdom

## Abstract

Azoospermia is one of the major reproductive disorders which cause male infertility in humans; however, the etiology of this disease is largely unknown. In the present study, six missense mutations of *WT1* gene were detected in 529 human patients with non-obstructive azoospermia (NOA), indicating a strong association between *WT1* mutation and NOA. The Wilms tumor gene, *Wt1,* is specifically expressed in Sertoli cells (SCs) which support spermatogenesis. To examine the functions of this gene in spermatogenesis, *Wt1* was deleted in adult testis using *Wt1^flox^* and *Cre-ER^TM^* mice strains. We found that inactivation of *Wt1* resulted in massive germ cell death and only SCs were present in most of the seminiferous tubules which was very similar to NOA in humans. In investigating the potential mechanism for this, histological studies revealed that the blood–testis barrier (BTB) was disrupted in *Wt1* deficient testes. In vitro studies demonstrated that *Wt1* was essential for cell polarity maintenance in SCs. Further studies found that the expression of cell polarity associated genes (*Par6b* and *E-cadherin*) and Wnt signaling genes (*Wnt4, Wnt11*) were downregulated in *Wt1* deficient SCs, and that the expression of *Par6b* and *E-cadherin* was regulated by *Wnt4*. Our findings suggest that *Wt1* is important in spermatogenesis by regulating the polarity of SCs via Wnt signaling pathway and that *WT1* mutation is one of the genetic causes of NOA in humans.

## Author Summary

Infertility is one of the most common health problems, affecting about 15% of the couples in the world. In about half of these couples, infertility is related to male reproductive defect. Azoospermia is one of the major causes of male infertility in humans. Previous studies have found that the mutation or deletion of some genes is associated with azoospermia; however, the genetic cause of this remains largely unknown. In the present study, we detected *Wt1* missense mutations in men with non-obstructive azoospermia (NOA). An essential function for WT1 in male spermatogenesis was confirmed by the use of a *Wt1* conditional knockout mouse strain. Inactivation of *Wt1* resulted in germ cell loss in mice, which was similar to NOA in human patients. Our data indicate that WT1 mutation is one genetic cause of male infertility and suggest that WT1 mutational analysis will be useful for diagnosis in a clinical setting.

## Introduction

Infertility is a common health problem which affects about 15–20% of couples. Among infertile couples, about 50% are related to male infertility [1], a major cause of which is azoospermia. Genetic causes of azoospermia include autosomal chromosome abnormalities, Y chromosome microdeletions, and single gene mutations. Several genes have been reported to play a role in azoospermia, including *PRM1, SPATA16, AURKC*, and *KLHL10* [2]–[5].

As support cells, Sertoli cell (SCs) play central roles in testis development and spermatogenesis. During mouse embryogenesis, SCs emerge at E10.5 and are involved in seminiferous cord formation and prevent germ-cell entry into meiosis [6]. Mullerian duct regression in males is induced by anti-Müllerian hormone (AMH) which is secreted by immature SCs [6], [7]. At puberty, SCs undergo terminal differentiation and develop complex morphological interactions with each other and with adjacent germ cells. Mammalian spermatogenesis is dependent on the proper functioning of SCs which provide structural support and nutrition to developing germ cells, phagocytose degenerating germ cells and residual bodies, release spermatids at spermiation, and produce a host of proteins that regulate or respond to pituitary hormone [8]–[10].

Our previous studies have demonstrated that *Wt1*, which encodes a nuclear transcription factor, is important for testes development with inactivation of *Wt1* in SCs between E12.5–E14.5, resulting in testicular cord disruption and testes dysgenesis [11]. However, the functional significance of *Wt1* in adult testis has been unclear, in part due to the gonadal agenesis of *Wt1^−/−^* [12] and *Wt1^R394W/R394W^* [13] mice. Rao et al reported that knock-down of *Wt1* using siRNA in postnatal SCs caused reduced sperm count [14], suggesting that *Wt1* plays a role in spermatogenesis. However, the exact function of *Wt1* in spermatogenesis and underlying mechanism by which it plays a role are still largely unknown.

In this study, we demonstrated that inactivation of *Wt1* in adult SCs resulted in massive germ cell death with only SCs surviving in the seminiferous tubules. Six *WT1* missense mutations were detected in 529 NOA patients by mutational analysis, indicating a strong association between *WT1* mutation and spermatogenic defects in human. We further demonstrated that *Wt1* is critical for maintaining the polarity of SCs, likely via Wnt signaling pathways. Inactivation of *Wt1* resulted in loss of polarity in SCs and abnormal tight junction assembly which in turn caused germ cell death.

## Results

### Inactivation of *Wt1* in adult testis results in massive cell death in seminiferous tubules

**Figure 1:**
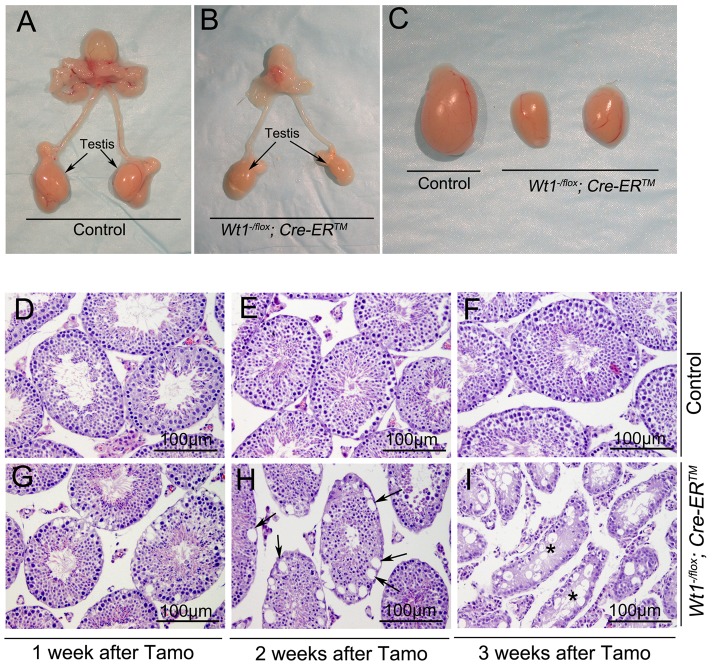
Inactivation of *Wt1* results in seminiferous tubule atrophy and testis hypoplasia. Reproductive tracts of control (A) and *Wt1^−/flox^; Cre-ER^TM^* (B) males 3 weeks after Tamoxifen-induced *Wt1* ablation. Compared to the control mice, the size of *Wt1^−/flox^; Cre- ER^TM^* testes is dramatically reduced (C). (D–I) Cross-sections of control and *Wt1^−/flox^; Cre- ER^TM^* testes at 1 (D, G), 2 (E, H), and 3 (F, I) weeks after Tamoxifen treatment. Vacuolization was detected in a small number of tubules at 1 week after Tamoxifen induction (G) and severe epithelial vacuolization (arrows) was noted at 2 weeks after Tamoxifen induction in *Wt1^−/flox^; Cre- ER^TM^* testis (H). Massive cell loss with empty tubules (asterisks) was observed in *Wt1^−/flox^; Cre- ER^TM^* testis at 3 weeks after Tamoxifen treatment (I).

*Wt1^−/flox^; Cre-ER^TM^* and control mice (*Wt1^−/flox^, Wt1^+/flox^; Cre-ER^TM^*) were obtained by intercrossing of *Wt1^flox/flox^* and *Wt1^+/−^; Cre-ER^TM^* mice. The growth of *Wt1^−/flox^; Cre-ER^TM^* mice were indistinguishable from that of control mice and the morphology and histology of *Wt1^−/flox^; Cre-ER^TM^* testes were completely normal (data not shown). To induce Cre activity, *Wt1^−/flox^; Cre-ER^TM^* and littermate control mice were injected with 9 mg/40 g (body weight) Tamoxifen for two consecutive days at 8 weeks of age. The testes were collected at 1, 2, and 3 weeks after Tamoxifen injection. The efficiency of Tamoxifen induced Cre recombination was examined by *Wt1* Real-time PCR (Figure S2G) and western blot (Figure S3). Compared to control testis *Wt1* mRNA was reduced about 50% in *Wt1^−/flox^; Cre-ER^TM^* testis, indicating that *Wt1* was deleted in about 50% of SCs. Because the Cre activation results in the in-frame deletion of exons 8 and 9, the Wt1Δ allele results in a truncated protein. Our previous work indicated that this truncation has the same phenotypic effect as Wt1 deletion [11]. As shown in Figure S3, approximately the same amount of wild type and truncated Wt1 protein was observed in *Wt1^−/flox^; Cre-ER^TM^* testes 1 week after Tamoxifen induction, indicating that Wt1 function was lost in approximately 50% of Sertoli cells. This was consistent with real time PCR results. The size of testes from *Wt1^−/flox^; Cre-ER^TM^* mice was dramatically reduced 3 weeks after Tamoxifen treatment (Figure 1C). Histological analysis results revealed that the seminiferous tubules were grossly normal in *Wt1^−/flox^; Cre-ER^TM^* testis 1 week after Tamoxifen induction (Figure 1G), although vacuolization was first noted in a small number of tubules. Severe epithelial vacuolization (Figure 1H, arrows) was noted in mutant testis 2 weeks after Tamoxifen induction. At 3 weeks post-Tamoxifen, atrophic seminiferous tubules with massive cell loss were evident in mutant testes (Figure 1I), whereas the control testis was completely normal (Figure 1F). In addition to germ cell loss, inactivation of *Wt1* in adult mice also resulted in multiple phenotypes, including accumulation of ascitic fluid (Figure S1A), atropic spleen (Figure S1B), abnormal pancreas (Fig. S1C), and renal failure (Figure S1D), consistent with a previous report [15]. In this study, however, the defect in spermatogenesis was not observed, likely because mice were treated with Tamoxifen for 5 consecutive days and, died earlier in our model system,

### *Wt1* is essential for germ cell survival

Apoptotic cells (Figure S4C, white arrows) were noted by TUNEL assay at the peripheral region of seminiferous tubules in *Wt1^−/flox^; Cre-ER^TM^* testis at 1 week after Tamoxifen induction, and the number of TUNEL positive cells (Figure S4D, white arrows) was dramatically increased at 2 weeks. In contrast, very few apoptotic cells were detected in control testis at both 1 (Figure S4A) and 2 weeks (Figure S4B). The results of statistical analysis showed that the number of apoptotic cells was significantly increased in *Wt1^−/flox^; Cre-ER^TM^* mice at both 1 and 2 weeks after Tamoxifen treatment compared to control testes (Figure S4E).

**Figure 2:**
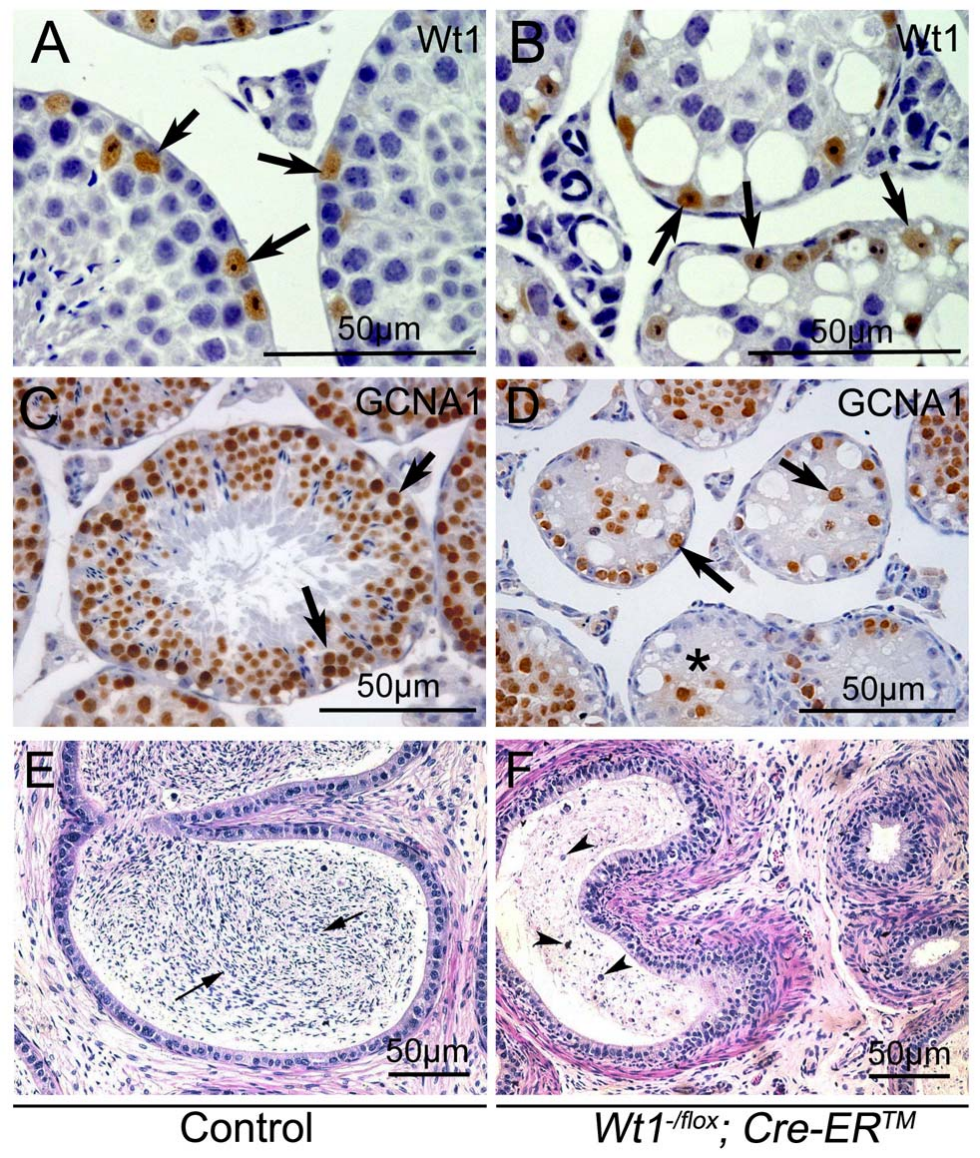
Loss of *Wt1* results in germ cell death: IHC and H & E staining of testes 3 weeks after Tamoxifen treatment. In control testes, the seminiferous tubules are filled with GCNA1 positive germ cells (C), whereas, very few GCNA1 positive germ cells (D, black arrows) are noted in the *Wt1^−/flox^; Cre- ER^TM^* testes and some tubules are completely void of germ cells (D, asterisks). *Wt1* positive Sertoli cells are present in both control (A) and *Wt1^−/flox^; Cre- ER^TM^* (B) testes. The cauda epididymes of control mice are filled with mature sperms (E, black arrows); in contrast, only immature spermatocyte and cell debris (arrow heads) are present in the cauda epididymes of *Wt1^−/flox^; Cre-ER^TM^* mice and no mature sperm are detectable (F).

To identify the cells which survived in *Wt1*-deficient testes, immunohistochemistry analyses were carried out. Because the truncated WT1 protein encoded by the recombined *Wt1^Δ^* allele is recognized by the Wt1 antibody, Wt1 antibody could be employed to identify Sertoli cells. *Wt1* positive Sertoli cells were localized at the periphery of the seminiferous tubules in both control (Figure 2A, arrows) and *Wt1^−/flox^; Cre-ER^TM^* (Figure 2B, arrows) testes. Multiple layers of GCNA1-positive germ cells were noted in seminiferous tubules of control testes (Figure 2C). However, very few GCNA1 positive germ cells were present in *Wt1^−/flox^; Cre-ER^TM^* testis (Figure 2D, arrows), and some tubules were complete devoid of germ cells (Figure 2D, asterisks). The cauda epididymes of control mice were filled with normal mature sperm (Figure 2E, arrows). In contrast, only cellular debris and round, prematurely released spermatocytes were noted in *Wt1^−/flox^; Cre-ER^TM^* mice (Figure 2F, arrow heads). These results indicate that *Wt1* is essential for SCs function such that *Wt1*-deficient SCs cannot support the development of germ cells, eventually resulting in germ cell death.

### *WT1* mutation is associated with azoospermia in humans

**Figure 3:**
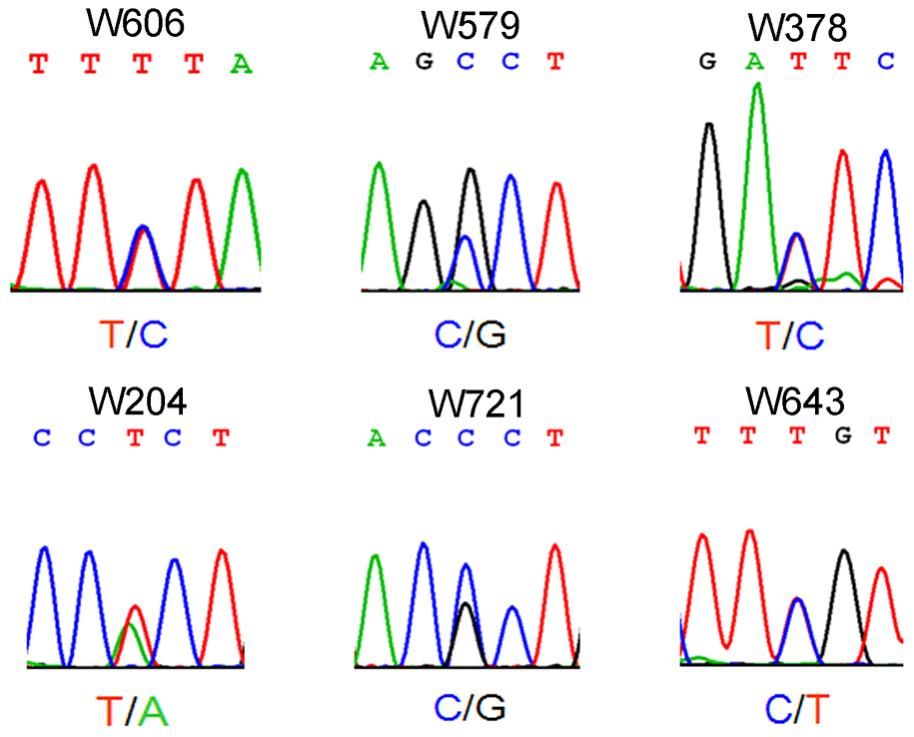
Electropherogram showing single-nucleotide mutation of *WT1* in patients with non-obstructive azoospermia.

Azoospermia is one of the major causes of male infertility in human. The testes histology of human non-obstructive azoospermia (NOA) was very similar to *Wt1*-deficient testes. As shown in Figure S6, only Sertoli cells (black arrows) were presented in the seminiferous tubules (asterisks) of human NOA patients. To determine whether *WT1* mutation is associated with spermatogenic defects in humans, following exonic capture we sequenced (mean depth of 43×) the exons of *WT1* in 529 non-obstructive azoospermia (NOA) patients and 709 men with proven fertility. On average per sample, 96% of target bases were covered at least once, and 78% were covered sufficiently for variant calling (Table S1). The first coding exon was poorly captured due to its extremely high GC content. As shown in Figure 3 and Table S2, 6 *WT1* missense mutations were detected in 6 patients while no mutations were found in the control group. By Chi-square analysis, there was a statistically significant (p = 0.004) difference in WT1 status between the NOA population and the control group. The WT1 variants present in the NOA patients have not been detected as SNPs in the 1000 Genomes Project, further indicating the significance of the observation. Two mutations were in the two zinc finger domains (encoded by exons 8 and 9) which are most important for DNA-protein interaction [16]. The other four mutations were localized to the transcription regulatory domain (Exons 3, 4, 6). The functional significance of the NOA-associated mutations was predicted using a combination of several approaches as previously described [17]. All 6 mutations were predicted to be deleterious by several complementary nsSNV scoring algorithms (Table S2), including SIFT [18], PolyPhen2 [19], PhastCons [20] and GERP scores [21] (Table S2). The observation of these predicted deleterious mutations specifically in the NOA population strongly indicates that *WT1* is also important for spermatogenesis in human and that *WT1* mutation plays an etiologic role in azoospermia.

### The integrity of the blood-testis barrier (BTB) is damaged in *Wt1*-deficient testes

**Figure 4:**
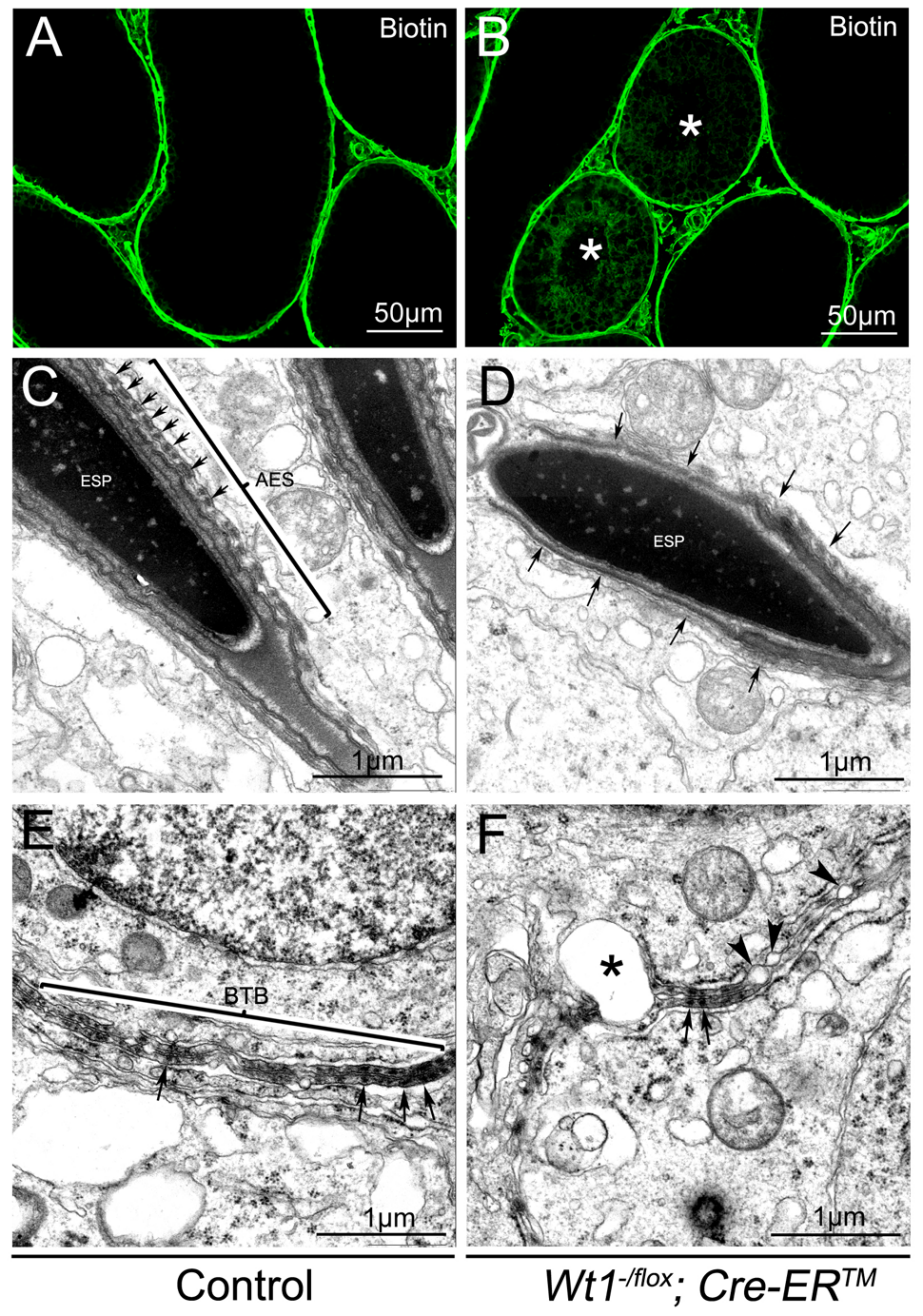
The integrity of the BTB is damaged in *Wt1^−/flox^; Cre-ER^TM^* testis 1 week after Tamoxifen treatment. In control testes, biotin tracer is restricted to the testicular interstitium and the basal compartment of the seminiferous tubules, with no tracer observed in the tubular lumen (A). Biotin tracer is detected along the Sertoli cell plasma membranes from the basement membrane to the lumen in some seminiferous tubules (asterisks) of *Wt1^−/flox^; Cre- ER^TM^* testes (B). By TEM a normal BTB structure (E) and apical ES ultrastructure with well-organized actin bundles(C, arrows) is observed in control testes, but *Wt1* mutant testes display abnormal apical ES structures (D, arrows) and the BTB structure is disrupted with numerous blisters (F, asterisks and arrow heads).

One of the major functions of SCs is to maintain the integrity of the BTB, which, when disrupted, results in germ cell death and spermatogenic defects [22], [23]. To test whether the integrity of the BTB was damaged in *Wt1*-deficient testes, surface biotinylated reagent was injected into the testicular interstitium of both control and *Wt1^−/flox^; Cre-ER^TM^* testes at 1 week after Tamoxifen treatment and before obvious histological changes were observed. In control mice, biotin tracer was restricted to the testicular interstitium and the basal compartment of the seminiferous tubules with no tracer being observed in the tubular lumen (Figure 4A, Figure S8A and C). However in Tamoxifen treated *Wt1^−/flox^; Cre-ER^TM^* testes, biotin tracer was also present along the SCs plasma membrane from the basement membrane to the lumen in about 30% of seminiferous tubules (Figure 4B, Figure S8B and D, asterisks). This difference was significant (Figure S8E). These results indicated that the integrity of BTB in *Wt1^−/flox^; Cre-ER^TM^* testes was disrupted after *Wt1* inactivation such that it was permeable to the biotin tracer.

The BTB structure in *Wt1*-deficient testes was further examined by transmission electron microscope (TEM). As shown in Figure 4E, a normal BTB (bracket) structure with F-actin bundles (black arrows) was observed in control testes. In contrast, in *Wt1*-deficient testes, the BTB was aberrant with intermittent loss of F-actin bundles (Figure 4F, black arrows) and the occurrence of vacuoles, likely formed as a result of loss of interaction between SCs (Figure 4F, asterisks and black arrowheads). We also found that the structure of apical ES (Ectoplasmic Specialization) was damaged in *Wt1^−/flox^; Cre-ER^TM^* testes at 1 week after Tamoxifen treatment. In control testes, the apical ES between Sertoli cell and elongated sperm was well organized with F-actin bundles (Figure 4C, arrows), whereas the apical ES structure was disrupted and there was a loss of F-actin bundles in *Wt1*-deficient testes (Figure 4D, arrows). The expression of BTB components was examined by immunofluorescence at 1 week after Tamoxifen treatment. As shown in Figure S7, tight junction protein Claudin11 (A, B), adhesion junction proteins N-cadherin (C, D) and β-catenin (E, F), and gap junction protein CX43 (G, H) were detected at the peripheral region of seminiferous tubules where tight junctions are formed in control testes (A, C, E, G). In contrast, the expression of these genes was significantly reduced in *Wt1^−/flox^; Cre-ER^TM^* testes (B, D, F, H), indicating the BTB structure was disrupted in *Wt1*-deficient testes at 1 week after Tamoxifen induction.

### Loss of epithelial cell morphology and tight junction formation in *Wt1*-deficient SCs

**Figure 5:**
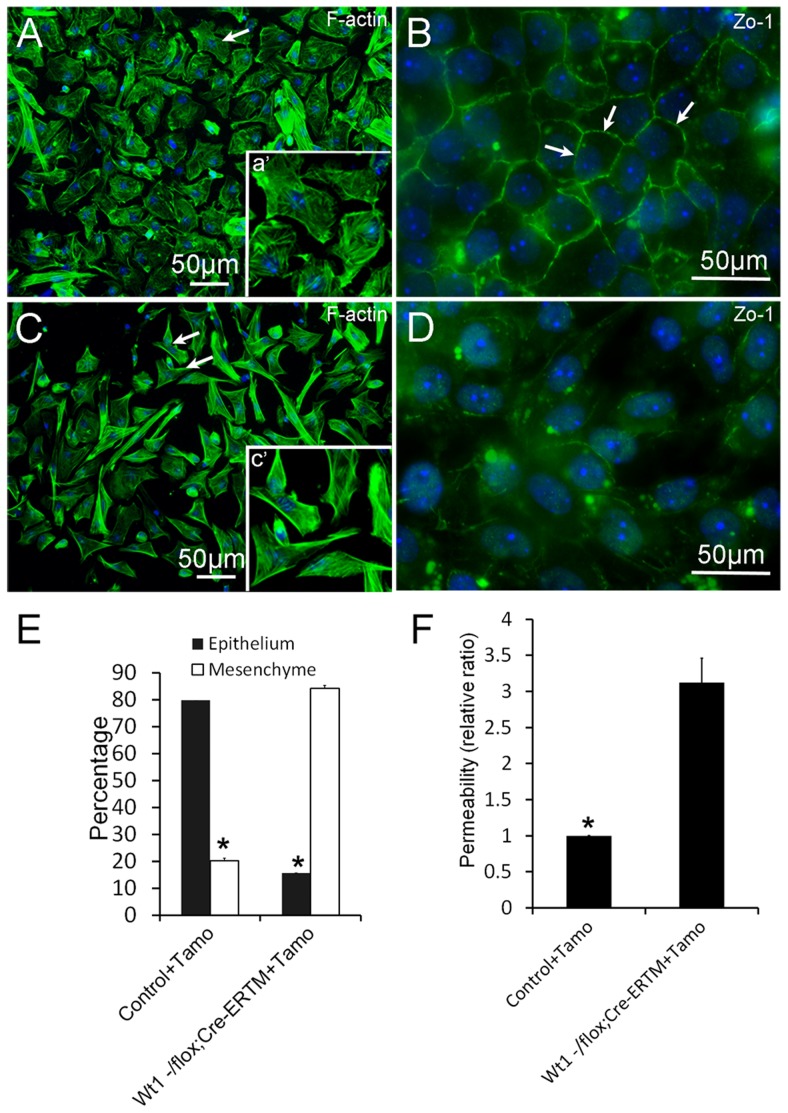
Deletion of *Wt1* causes morphologic changes and loss of tight junction formation in cultured SCs. Control SCs display a typical epithelial morphology (A and E), while *Wt1*-ablated SCs display a mesenchyme-like morphology (C and E). The tight junctions between SCs were assessed by ZO-1 staining. ZO-1 protein is present along the boundary between control SCs (B, white arrows). In contrast, ZO-1 protein is diffused in the cytosol and not detected along the cell boundary in *Wt1*-deficient SCs (D). (F) FITC-dextran flux assays indicated increased permeability in *Wt1*-deficient SCs compared to control SCs. * *p*<0.05.

To explore the physiological function of *Wt1* in spermatogenesis, control and *Wt1^−/flox^; Cre-ER^TM^* SCs were isolated from 2 months old mice and cultured *in vitro*. *Wt1* was deleted by addition of 4-OH-tamoxifen to the culture medium. As shown in Figure 5A, the control SCs had a cuboidal epithelial morphology as visualized by F-actin staining (Figure 5A). In contrast, SCs assumed a mesenchymal-like morphology when *Wt1* was inactivated by Tamoxifen induction (Figure 5C). The quantitative results showed that the number of mesenchymal-like cells was dramatically increased after Wt1 inactivation, and this difference was significant (Figure 5E). Tight junctions were well established in control SCs after a few days culture indicating by *ZO-1* staining (Figure 5B, white arrows). However, in *Wt1*-deficient SCs, *ZO-1* protein was diffused in the cytosol and no obvious staining was noted at the cell junctions (Figure 5D). The tight junction formation by SCs was further examined by Paracellular FITC-dextran Flux assay. As shown in Figure 5F, the permeability of *Wt1*-deficient SCs was significantly increased compared to control cells. This result further confirmed that the tight junction formation of SCs is disrupted when *Wt1* is inactivated, consistent with the *Zo-1* staining data.

### The expression of SCs specific genes is not changed in Tamoxifen treated *Wt1^−/flox^; Cre-ER^TM^* testes and *Wt1*-deficient SCs

Our previous study found that deletion of *Wt1* in SCs during early stage of embryonic development resulted in downregulation of Sox9 and AMH [11]. To test whether deletion of *Wt1* in adult testes also affects the expression of SC specific genes, immunohistochemistry and real time PCR were conducted. The results of immunohistochemistry showed that the expression of Sox9 (Figure S2A, B), AR (Figure S2C, D), and Gata4 (Figure S2E, F) was not changed in *Wt1^−/flox^; Cre-ER^TM^* testes (Figure S2B, D, F) 3 weeks after Tamoxifen treatment. Real time PCR further confirmed these results (Figure S2G). The expression of SC-specific genes was also examined in *Wt1*-deficient SCs by immunofluorescence and real time PCR. As shown in Figure S9, inactivation of *Wt1* in cultured primary SCs did not affect the expression of SC specific genes, such as *Sox9, Dmrt1, Gata4, AR, Gata1*, and *Nr5a1*.

### Molecular characterization of spermatogenic defect in *Wt1*-deficient testis

To explore the molecular mechanism of the spermatogenic defect in *Wt1*-deficient testes, RNA-Seq analysis were performed using mRNA from control and *Wt1*-deficient SCs. A total of 710 differentially (p-value<0.05) expressed genes were identified (456 upregulated and 254 downregulated genes), the raw data has been uploaded to http://www.ncbi.nlm.nih.gov/geo/, the accession number is GSE46664. As listed in Table S3, the genes were differentially expressed in multiple pathways based on the pathway term analysis. However, no specific pathway was significantly altered in *Wt1*-deficient SCs. In the present study, we found that the morphology of SCs was transformed from epithelium into mesenchyme with a concurrent loss tight junction formation after *Wt1* inactivation. It has been reported previously that *Wt1* is involved in EMT process during kidney and heart development [24], [25]. Therefore, EMT and cell polarity related genes were selected for further analysis. RNA-Seq analysis revealed that cell polarity-associated genes, such as *E-cadherin, Par6b*, were significantly decreased in *Wt1*-deficient SCs. In contrast, the expression of the EMT related genes was not significantly changed. We also found that Wnt signaling gene, *Wnt11* was downregulated in *Wt1*-deficient SCs. The expression of *Wnt4* was also decreased in *Wt1*-deficient SCs, but this was not statistically significant.

**Figure 6:**
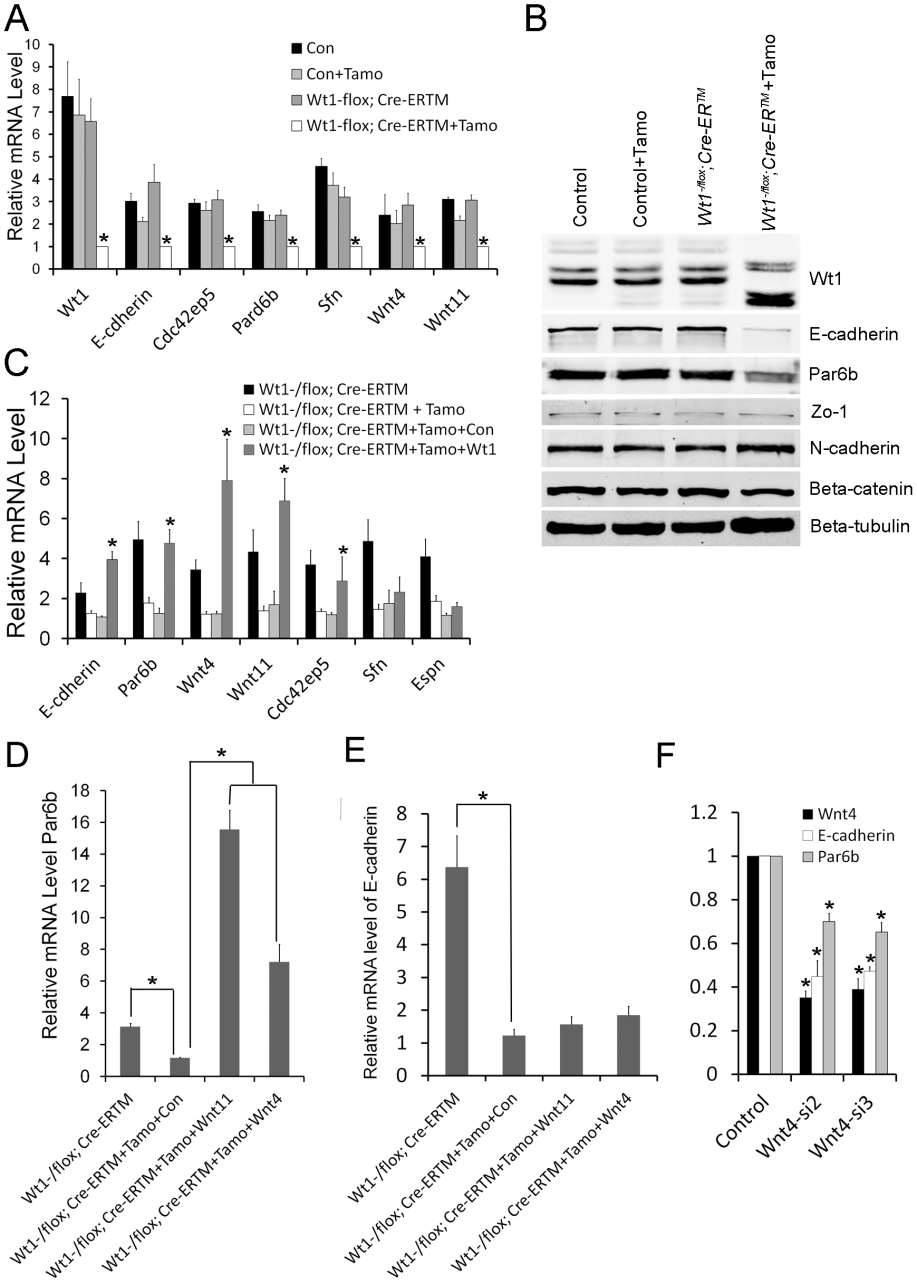
Real time PCR and Western blot verification in SC cultures of altered expression of cell polarity and Wnt signaling genes following *Wt1* ablation. (A) The expression of cell polarity related genes (*E-cadherin, Par6b, Cdc42bp5, Sfn*) and Wnt signaling genes (*Wnt4* and *Wnt11*) is significantly decreased in *Wt1*-deficient SCs (white bars). (B) E-cadherin and Espin protein expression is significantly reduced in *Wt1*-deficient SCs, whereas the expression of *occludin*, *N-cadherin, Zo-1*, and *β-catenin* is not changed. (C) The expression of *E-cadherin, Par6b, Wnt4, Wnt11*, and *Cdc42ep5* in *Wt1*-deficient SCs was rescued by transfection with *Wt1* expressing adenovirus. (D) The expression of *Par6b* was induced by *Wnt4* and *Wnt11* expressing adenovirus in *Wt1*-deficient SCs. (E) The expression of *E-cadherin* was not rescued by *Wnt4* and *Wnt11* expressing adenovirus in *Wt1*-deficient SCs. (F) *Wnt4* was knocked down by two different siRNAs (siWnt4-2 and siWnt4-3) in SCs and the expression of *E-cadherin* and *Par6b* was significantly decreased compared to control cells (NC) treated with a scrambled siRNA. * *p*<0.05.

To further verify the RNA-Seq results, the expression of genes related to EMT and cell polarity was analyzed by real time PCR. As shown in Figure 6A, the expression of *Par6b, E-cadherin, Wnt11*, and *Wnt4* was significantly decreased in *Wt1*-deficient SCs. Western blot analysis also showed that the expression of E-cadherin and Par6b was dramatically reduced in *Wt1*-deficient SCs, however, ZO-1 and N-cadherin expression was not changed (Figure 6B). In contrast, *Snail1, Snail2, Twist, Zeb, Sip, Mmp2* were not differentially expressed between control and *Wt1*-deficient SCs (Figure S10). The expression of cell polarity related genes in *Wt1*-deficient testes was also examined by immunofluorescence and real time PCR. The results of immunofluorescence (Figure S5A–D) showed that the localization of *E-cadherin* and *Par6b* was disorganized in *Wt1^−/flox^; Cre-ER^TM^* testes at 1 week after Tamoxifen induction. Real time PCR results showed that the mRNA level of *Par6b* and *Wnt4* was significantly reduced in *Wt1^−/flox^; Cre-ER^TM^* testes at 1 week after Tamoxifen treatment. mRNA level of *E-cadherin* and *Wnt11* was also reduced, but this was not statistically significant (Figure S5E). These results were consistent with the results from the *in vitro* study. Notably, when Tamoxifen treated *Wt1^−/flox^; Cre-ER^TM^* SCs were transfected with *Wt1* expressing adenovirus, the mRNA level of *Par6b, E-cadherin, Wnt4*, and *Wnt11* was completely rescued, indicating that the expression of these genes was regulated by *Wt1* directly or indirectly (Figure 6C).

### The expression of *E-cadherin* and *Par6b* is regulated by *Wnt4* in SCs

To examine whether *Wt1*'s role in regulating SCs polarity is mediated by Wnt signaling, *Wnt4* was knocked down in cultured SCs using siRNA. As shown in Figure 6F, compared to treatment with scrambled siRNA, the mRNA level of *Wnt4* was reduced about 70% after transfection with *Wnt4*-specific siRNA, and the expression of *E-cadherin* and *Par6b* was also reduced about 30–50% respectively (Figure 6F), suggesting that the expression of *E-cadherin* and *Par6b* is regulated by *Wnt4* in SCs. To further confirm these results, Tamoxifen treated *Wt1^−/flox^; Cre-ER^TM^* SCs were transfected with *Wnt4*- and *Wnt11*- expressing adenovirus. We found that the expression of *Par6b* was dramatically increased upon over-expression of both *Wnt4* and *Wnt11* in *Wt1*-deficient SCs (Figure 6D). However, the expression of *E-cadherin* was not rescued (Figure 6E), suggesting that the expression of E-cadherin was also directly regulated by *Wt1*.

### The mutations detected in NOA patient results in WT1 protein loss of function

**Figure 7:**
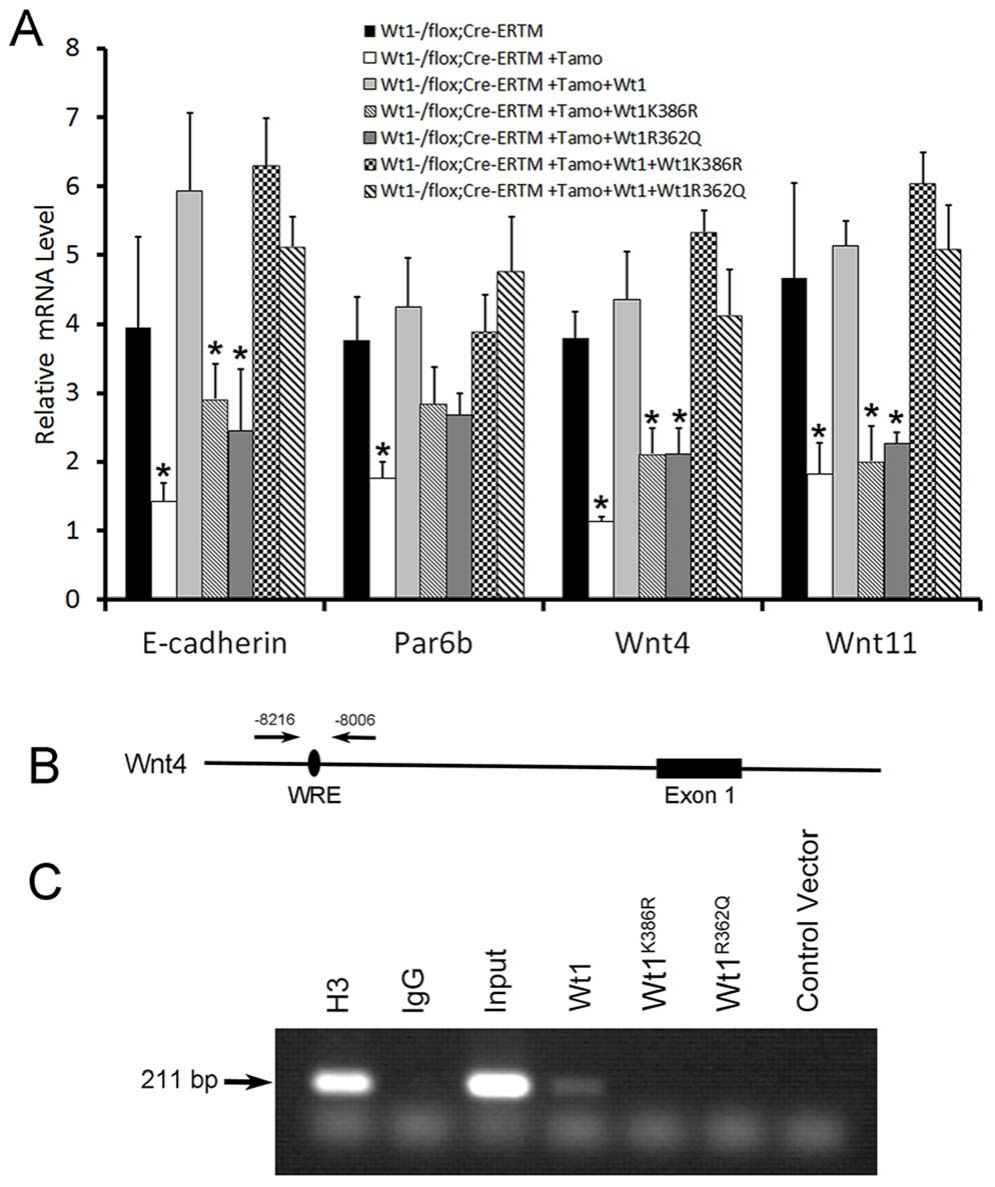
Functional analysis of WT1 mutations detected in NOA patients. (A) The expression of *Wnt4, Wnt11*, and *E-cadherin* in *Wt1*-deficient SCs was rescued by wild type *Wt1* expressing adenovirus, but not *Wt1^R362Q^* and *Wt1^K386R^* expressing adenovirus. Co-transfection of *Wt1^R362Q^* and *Wt1^K386R^* in *Wt1*-deficient SCs did not affect *Wt1* induced *Wnt4, Wnt11*, and *E-cadherin* expression. (B) Schematic image of *Wnt4* promoter, black oval indicated the predicted *Wt1* binding site. (C) A 211 bp band in *Wnt4* promoter was amplified after pulled down with Wt1 antibody in *Wt1* expressing adenovirus transfected HepG2 cells, but not in *Wt1^R362Q^* and *Wt1^K386R^* expressing adenovirus transfected HepG2 cells. Histone3 antibody and input was used as positive control, and IgG and vector was used as negative control.

Although the *WT1* mutations detected in NOA patients were predicted to be deleterious by several complementary nsSNV scoring algorithms (Table S2), the effect of mutations on *Wt1* function was further assessed by in vitro functional analyses. Two *Wt1* expressing adenovirus carrying the zinc finger domain mutations detected in patient W643 and W606 were generated by site-directed mutagenesis and designated as *Wt1^R362Q^* and *Wt1^K386R^*. We found that both of these mutations did not affect the nuclear localization of Wt1 protein (Figure S11). However, functional analysis showed that both these mutated Wt1 could not induced the expression of *E-cadherin, Par6b, Wnt4*, and *Wnt11* in *Wt1*-deficient SCs (Figure 7A), indicating that these mutations caused Wt1 protein loss of function. To examine whether these mutation affect the ability of Wt1 protein binding to the promoter of a Wt1 targeting gene, a ChIP assay was performed using HepG2 cells. As shown in Figure 7B, C, a 211 bp DNA fragment in the *Wnt4* promoter region was pulled down by Wt1, but not Wt1^R362Q^ and Wt1^K386R^. These results indicated that these mutations affected the interaction between Wt1 protein and DNA.

## Discussion

*Wt1* is specifically expressed in SCs of the testes and our previous study has demonstrated that it is critical for testicular development [11], [12]. However, the functional significance of *Wt1* in adult testes is largely unknown. In the present study, we have demonstrated for the first time *in vivo*, using a conditional *Wt1* knockout mice strain, that *Wt1* plays an important role in maintaining SC polarity and that inactivation of *Wt1* leads to the loss of epithelial characteristics of SCs which in turn results in germ cell death.

In adult mammalian testes, SCs are polarized epithelial cells which, as nurse cells, are essential for germ cell division and differentiation [26], providing structural support and creating an immunological barrier from the systemic circulation [26], [27]. The latter function is conferred by the blood-testes barrier (BTB), which is formed by tight junctions (TJs), basal ectoplasmic specialization (ES), and desmosome-like junctions between SCs [23], [28]. The integrity of the BTB structure is very important for spermatogenesis, and abnormal assembly of BTB is known to cause germ cell loss and male infertility [29].

In this study, we have demonstrated, both by biotin tracer injection experiments (Figure 4B) and TEM (Figure 4F), that loss of *Wt1* disrupted the normal BTB structure. While *Wt1* is a transcriptional regulator, the expression of genes known to be important in spermatogenesis, *AR* [30], *Dmrt1* [31], *Gata4* [32], *Connexin43* [33], *Claudin11* [34], was not changed in *Wt1*-deficient testes, suggesting that the spermatogenic defect of *Wt1*-deficient mice was mechanistically different.

Analysis of cultured SCs revealed that upon *Wt1* deletion, cells underwent a morphologic transformation from cuboidal epithelium into mesenchyme-like cells with a concomitant loss of tight junctions and a significant reduction in E-cadherin expression, both of which are normal features of epithelial cells. These results indicate that *Wt1* is critical for the maintenance of epithelial characteristics of SCs and that the loss of these characteristics results in germ cell death.

A recent study has shown that *Wt1* is essential for epithelial to mesenchymal transition (EMT) in epicardial cells by controlling the expression of *Snai1* [25]. However, we found that the expression of the EMT-related genes (such as *Snail, Twist, Zeb, Sip*, et al) was not changed in *Wt1*-deficient SCs. Instead, genes important for cell polarity maintenance (such as *Par6b, Cdc42ep5*) and Wnt pathway signaling genes (*Wnt4* and *Wnt11*) were significantly decreased in SCs when *Wt1* gene was deleted. These results suggest that the function of *Wt1* is different in different cell types which is probably dependent on the interaction with different cofactors.

PAR6b directly binds to PAR3, aPKC, and CDC42 [35]. This complex is involved in tight junction formation in epithelial cells [35], and inhibiting the expression of *Par3*, *Par6*, or *aPKC* blocks tight junction assembly in MDCK cells [35], [36]. The PAR3/PAR6b/aPKC complex is also involved in regulating apical ectoplasmic specialization (ES) and blood testes barrier (BTB) restructuring in the testis. Knockdown of *Par6b* or *Par3* results in defective tight junction formation in cultured SCs [37]. These data suggest that the spermatogenesis defect we observed following *Wt1* ablation is due to the loss of SC polarity.

Non-canonical Wnt signaling is known to play an important role in regulating cell polarity and motility [38]. In vertebrates, *Wnt4*, *Wnt5a* and *Wnt11* encode ligands that activate the Wnt signaling pathway [39]–[41] that is critical for regulating epithelial versus mesenchymal cell characteristics. *Wnt4* is necessary and sufficient for MET during kidney development [42], [43], and *Wt1* directly activates *Wnt4* expression in developing kidney. Interestingly, by recruiting different cofactors, Wt1 represses *Wnt4* expression and induces an epithelial to mesenchymal transition in epicardium [24]. In this study, we found that the expression of *Wnt4* and *Wnt11* was activated by *Wt1* in SCs and that deletion of *Wt1* resulted in downregulation of these genes and loss of epithelial cell polarity. *Wnt4* RNAi knockdown experiments showed that the expression of *Par6b* and *E-cadherin* was regulated by *Wnt4* in SCs. On the other hand, overexpression of *Wnt4* and *Wnt11* in *Wt1*-deficient SCs induced *Par6b* expression. These data suggest that *Wt1* controls SCs polarity indirectly through *Wnt4*. However, studies in 3T3 cells suggest that *Wt1* can directly regulates *E-cadherin* expression [44], and our data cannot exclude this possibility in SCs.

*WT1* germline mutation is known to result in a predisposition to Wilms tumors, male sex differentiation disorder, and early-onset renal failure [45]–[51]. These *WT1* mutations are truncating mutations or missense mutations that are predicted, based on structural studies, to alter the ability of zinc finger domains to bind to DNA. We have now identified 6 novel missense mutations in NOA patients. These data strongly suggest that *WT1* is also important for spermatogenesis in humans and that *WT1* gene mutations play an etiologic role in azoospermia. Four of these mutations occur in the regulatory domain of the protein. Two occur in exons encoding zinc finger domains known to be critical for DNA binding, and functional studies revealed that they affected the ability of Wt1 to bind to *Wnt4* promoter and result in a failure to induce targeting gene expression. Of not, these Wt1 mutants did not act in a dominant negative manner.

Interestingly, neither aberrant sex determination nor renal failure was noted in the NOA patients carrying *WT1* mutations. We speculate that the NOA-associated mutations represent a functionally new type of *WT1* alteration which probably also affects the ability to bind other transcription factors and/or chromatin-remodeling factors critical for its regulatory role.

In summary, our data strongly support a model by which loss of *Wt1* in SCs results in downregulation of non-canonical Wnt signaling genes (*Wnt4* and *Wnt11*) and cell polarity genes (*E-cadherin* and *Par6b*). This altered expression subsequently leads to loss of the epithelial characteristic and BTB integrity in SCs and concomitant germ cell loss. Such disruption of the BTB is also known to result in germ cell loss and male infertility in humans, and our observation of *WT1* mutations in NOA patients strongly suggests that altered WT1 function constitutes one genetic cause of azoospermia in humans. Wt1 mutational analysis will be potentially useful clinically for characterizing NOA patients.

## Materials and Methods

### Patient samples

Peripheral blood samples from 529 patients with non-obstructive azoospermia (NOA) and 709 men with proven fertility were collected from Peking University Shenzhen Hospital and the Center of Reproductive Medicine, Tongji Medical College, Huazhong University of Science and Technology. The inclusion criteria for NOA patients included: 1) no sperm detected in the pellets of semen samples on three different occasions, 2) no inflammation and injury of the reproductive system or pelvic cavity, 3) no endocrinological defect, and 4) no karyotypic abnormality nor Y chromosome microdeletion. Testicular biopsy and histological analysis were conducted for azoospermic men wherever possible. All the control men had fathered at least one child. This study was approved by the ethical committees of Peking University Shenzhen Hospital and Tongji Medical College, and all participants signed the consent form permitting the collection and use of their blood samples in the study.

### Selective exon capture and massively parallel sequencing

Genomic DNA was isolated from the blood samples using QIAamp DNA Mini Kits (QIAGEN). The exons of *WT1* and other 600 genes were selectively captured by NimbleGen custom arrays (Roche NimbleGen, Inc, USA) and sequenced following the standard Illumina-based resequencing procedures as described [52]. Reads were mapped to the UCSC human reference genome build hg19 by SOAPaligner [53]. Mutation analysis for all the exons of *WT1* were performed with SOAPsnp [54]. Novel coding mutations in *WT1* were defined as those variants that had not been annotated in dbSNP nor in the publicly available dataset from the 1000 Genome Project. To further refine those novel mutations that may be associated with NOA, all the genetic variants detected in the fertile men were also eliminated for subsequent analysis.

### Validation of novel mutations by Sanger sequencing

To validate the novel mutations identified in the *WT1* gene by the massively parallel sequencing, primers flanking the point mutations were designed with Primer3 software (primer sequences are given in Table S5). PCR was performed using the following conditions: 94°C for 7 minutes; 30 cycles of denaturation at 94°C for 30 seconds, annealing at 57–60°C for 45 seconds, extension at 72°C for 1 minute, and a final extension at 72°C for 7 minutes. PCR products were checked on 1.5% agarose gel. The amplification product was directly sequenced using the 3730 DNA analyzer (Applied Biosystems).

### Mice

All animal work was carried out in accordance with institutional animal care and use committee (IACUC) regulations. All the mice were maintained in a C57BL/6;129/SvEv mixed background. *Wt1^+/flox^* [11] mice were mated with mice carrying the *Wt1*-null allele (*Wt1^+/−^*) [12] and *Cre-ER^TM^* [55] transgenic mice to produce *Wt1^−/flox^; Cre-ER^TM^* offspring. DNA isolated from tail biopsies was used for genotyping. Genotyping was performed by PCR as described previously [11], [56].

### Tamoxifen injection

Tamoxifen (Sigma) was dissolved in corn oil at a final concentration of 20 mg/ml. Two month old control (*Wt1^+/flox^; Cre-ER^TM^*, *Wt1^−/flox^, and Wt1^+/flox^*) and *Wt1^−/flox^; Cre- ER^TM^* males were injected intraperitoneally with 9 mg/40 g body weight for 2 consecutive days.

### Tissue collection and histological analysis

Testes were dissected from mutant and control mice immediately after euthanasia and fixed in 4% paraformaldehyde for up to 24 hr, stored in 70% ethanol, and embedded in paraffin. Five-micrometer-thick sections were cut and mounted on glass slides. After deparaffinization, slides were stained with H&E for histological analyses.

### Immunohistochemistry analysis

IHC analysis of tissues from at least three mice for each genotype was performed using a Vectastain ABC (avidin–biotin–peroxidase) kit (Vector Laboratories, Burlingame, CA) as recommended and using antibodies to WT1 (Santa Cruz, sc-192) and GCNA1 (gift of Dr. George Enders). The IHC procedure was performed as described previously [13]. Stained slides were examined with a Leica DMR Epifluorescence Microscope, and images were captured by a Hamamatsu CCD camera.

### RNA-Seq analysis

Total RNA was prepared from cultured SCs isolated from 2 month old control (*Wt1^+/flox^; Cre-ER^TM^, Wt1^−/flox^, and Wt1^+/flox^*) and *Wt1^−/flox^; Cre-ER^TM^* mice after Tamoxifen treatment using an RNeasy Minikit (Ambion, Austin, TX). The main reagents and instruments used for RNA library construction and deep sequencing were the Illumina Gene Expression Sample Prep Kit, Solexa Sequencing Chip (flowcell), Illumina Cluster Station and Illumina HiSeq 2000 System. Sequence tags were prepared using the Illumina Digital Gene Expression Tag Profiling Kit, according to the manufacturer's protocol. The RNA-Seq was performed as described by Zhang et al [57]. In brief, raw data was filtered to remove adaptor tags, low quality tags and tags with a single copy number. Clean tags were classified according to their copy number and the saturation of the library was analyzed. All clean tags were mapped to the reference sequences, filtered and the remainder of the clean tags was designated as unambiguous clean tags. The number of unambiguous clean tags for each gene was calculated and normalized to the number of transcripts per million clean tags (TPM). To identify DEGs between control and Wt1-deficient Sertoli cells, the number of raw clean tags in each library was normalized to the TPM to obtain the normalized gene expression level. DEGs was identified as previously described [58] using a false discovery rate (FDR)≤0.001 and a threshold absolute log2-fold change ≥1 for the sequence counts across the libraries.

### Primary Sertoli cell isolation

A modified method was used to isolate primary Sertoli cells from the testes of 6-week-old mice [59]. Testes were decapsulated under the dissection microscope. The seminiferous tubules were pooled and washed with phosphate-buffered saline (PBS) three times. The tubules were incubated with 2 mg/ml collagenase I (Sigma) and 0.5 mg/ml DNase I (sigma) in DMEM for 30 minutes at 37°C on a shaker, then washed twice with DMEM and further digested with 2 mg/ml collagenase I, 0.5 mg/ml DNase I and 1 mg/ml hyaluronidase type III (Sigma) for 20–30 minutes at 37°C. The tubules were allowed to settle and were then washed twice with DMEM before being digested with 2 mg/ml collagenase I, 0.5 mg/ml DNase I, 2 mg/ml hyaluronidase, and 1 mg/ml trypsin for 40–60 minutes at 37°C. This final digestion step resulted in a cell suspension containing primarily Sertoli cells and type A spermatogonia. The dispersed cells were then washed twice with DMEM and placed into culture dishes in DMEM containing 10% fetal calf serum and incubated at 37°C and 5% CO2. Spermatogonia were unable to attach to the dish and were removed after the medium change on the next day. 4-OH-Tamoxifen (Sigma, H7904) was dissolved in ethanol to generate a 1 mM stock solution and further diluted to appropriate concentrations prior to use. Recombination was initiated by adding 4-OH-TM to cultured Sertoli cells at a final concentration of 1 µM. After 3 days culture, total RNA and protein were extracted as described below.

### Nucleic acid isolation and quantitative reverse transcription-PCR

Total RNA was extracted from cultured Sertoli cells or testes using a Qiagen RNeasy kit in accordance with the manufacturer's instructions. To quantify gene expression, real-time SybrGreen assay was performed with the isolated RNA. Gene expression was quantified relative to the expression of the gene for *Gapdh* (glyceraldehyde-3-phosphate dehydrogenase). Primers used for the RT-PCR are listed in sTable 4.

### Western blotting

Cells were lysed in radioimmune precipitation assay lysis buffer (RIPA) containing complete Mini protease-inhibitor cocktail tablets (Roche, Mannheim, Germany). The protein concentration in the supernatants was estimated using a Bradford assay (Bio-Rad Laboratories, Hercules, CA). The proteins were electrophoresed under reducing conditions in 12% SDS-PAGE gels and transferred to nitrocellulose membranes. Blots were incubated overnight at 4°C with primary antibody and followed by 1 h of incubation at room temperature with HRP-labeled secondary antibody. Specific signals were detected using the ECL Western blotting detection system.

### Biotin tracer

The permeability of the BTB was assessed by using a biotin tracer ENREF-37 [22]. Two-month-old control and *Wt1^−/flox^; Cre- ER^TM^* animals were injected with 9 mg/40 g body weight of tamoxifen. A week later they were anesthetized with avertin, and 50 µl of 10 mg/ml EZ-Link Sulfo-NHS-LC-Biotin (Pierce Chemical Co.) freshly diluted in PBS containing 1 mM CaCl2 was injected into the interstitium of one testis and the other testis was injected with 50 µl of 1 mM CaCl2 in PBS as an internal control. The animals were euthanized 30 min later, and the testes were removed immediately and embedded with OCT. Cryosections were prepared for further staining.

### Electron microscopy assay

Testes were collected from control and *Wt1^−/flox^; Cre-ER^TM^* males 1 week after Tamoxifen treatment and fixed overnight in 2.5% glutaraldehyde in 0.1 M phosphate buffer (pH 7.4). They were then washed in phosphate buffer (two changes), postfixed with 1.0% osmium tetroxide, dehydrated in a graded series of ethanol, and embedded in EPON/Araldite resin. Thin sections were cut, mounted on 200-mesh grids, and stained with uranyl acetate and lead citrate.

### Site-directed mutagenesis and generation of adenovirus

Mutant *Wt1* cDNA was generated using the QuikChange Site-Directed Mutagenesis Kit (Stratagene, La Jolla, CA). Mouse *Wt1* cDNA (-KTS) was cloned by our lab previously. The WT1 mutations detected in patients W643 and W606 caused amino acid changed from Arg to Gln and Lys to Arg respectively (Table S2). We generated the same mutations with mouse *Wt1* cDNA using the following primers (*Wt1^R362Q^*: forward, 5′CTTCAAGGACTGCGAGCAAAGGTTTTCTCGCTCAG3′, reverse, 5′CTGAGCGAGAAAACCTGTTCTCGCAGTCCTTGAAG3′; *Wt1^K386R^*: forward, 5′ CATTCCAGTGTAGAACTTGTCAGCG3′, reverse, 5′ CGCTGACAAGTTCTACACTGGAATG3′). The adenovirus containing wild type *Wt1*, *Wt1^R362Q^*, and *Wt1^K386R^* cDNA were generated using the Gateway Expression System (Invitrogen). The candidate genes were amplified by PCR and inserted into the pEntr 3C vector (Invitrogen). The resulting plasmids were then generated by homologous L/R recombination. Viral constructs were transduced into a 293A cell line, and high titer (10^8^ IU/ml) viral particles were obtained by 4 rounds of amplification. The titer of virus was determinated as previous described [60].

### ChIP assay

Chromatin immunoprecipitation (ChIP) assays were performed according to the protocol provided by Upstate Biotechnology (Charlottesville, VA). In brief, HepG2 cells were transiently transfected with *Wt1*, *Wt1* ^R362Q^, and *Wt1* ^K386R^ expressing adenovirus. 72 hr after transfection, cells were crosslinked with 1% formaldehyde in medium at 37°C for 15 min. Cells were then washed in ice-cold phosphate-buffered saline (PBS) and resuspended in 200 µl of SDS lysis buffer containing protease inhibitor mixture. The suspension was sonicated on ice and pre-cleared with protein A-agarose beads blocked with sonicated salmon sperm DNA (Upstate Biotechnology) for 30 min at 4°C. The beads were removed, and the chromatin solution was immunoprecipitated with anti-Wt1 antibody at 4°C, followed by incubation with protein A-agarose beads for an additional 1 h at 4°C. Normal rabbit IgG was used as a negative control, anti-Histone antibody was used as a positive control. The immune complexes were eluted with 100 µl of elution buffer (1% SDS and 0.1 M NaHCO_3_), and formaldehyde cross-links were reversed by heating at 65°C for 6 h. Proteinase K was added to the reaction mixtures and incubated at 45°C for 1 h. DNA of the immunoprecipitates and control input DNA were purified and then analyzed by standard PCR using mouse *Wnt4* promoter specific primers (Forward, 5′ ATAGCAAGCTCATGTGGTGTGCAG3′, reverse, 5′ ATATAGGCCGCCGCACTTATCAGA3′).

### Statistical analysis

Experiments were repeated at least three times. The data were evaluated for statistical differences using student T-test. A p-value<0.05 was considered significant.

## Supporting Information

Figure S1 Inactivation of *Wt1* results in multiple organ defects. (A) 3 weeks after Tamoxifen treatment, ascitic fluid was noted in *Wt1*-deficient mice (right). (B) Images of atropic spleen (left) in *Wt1*-deficient mice compared with control spleen (right). (C) Abnormal pancreas (upper) from *Wt1*-deficient mice compared to pancreas from control mice (lower). (D) Severe glomerulosclerosis in *Wt1*-deficient mice.
(TIF)

Figure S2 The expression of Sertoli cells-specific genes is not changed in *Wt1*-deficient testes. The expression of *Sox9* (A,B), *AR* (C, D), and *Gata4* (E, F) in Sertoli cells of control (A, C, E, arrows) and *Wt1^−/flox^; Cre-ER^TM^* (B, D, F, arrows) testes at 3 weeks after Tamoxifen treatment was analyzed by immunohistochemistry, and no obvious difference was observed between control and *Wt1*-deficient mice. (G) The mRNA level of *AR, Dmrt1, Gata4*, and *Sox9* in control and *Wt1^−/flox^; Cre-ER^TM^* testes at 1 week after Tamoxifen treatment was analyzed by real time PCR.
(TIF)

Figure S3 Western blot results of Wt1 expression in control and *Wt1^−/flox^; Cre-ER^TM^* testes at 1 week after Tamoxifen treatment, showing expression of the truncated Wt1 protein following deletion of exon 8 and 9.
(TIF)

Figure S4 Apoptotic cells are detected in *Wt1^−/flox^; Cre- ER^TM^* testis after Tamoxifen treatment. Very few TUNEL-positive cells (green, white arrows) were observed in control testes at 1 (A) and 2 (B) weeks after Tamoxifen induction. TUNEL positive cells (green, white arrows) were observed in *Wt1^−/flox^; Cre- ER^TM^* testis at 1 week after Tamoxifen treatment (C), and the number of apoptotic cells (green, white arrows) was dramatically increased 2 weeks after Tamoxifen treatment (D). (E) The results of statistical analysis showed that the difference between control and *Wt1*-deficient testes was significant. * *p*<0.05.
(TIF)

Figure S5 Immunofluorescence of E-cadherin and Par6b and RT-PCR results. The expression of E-cadherin and Par6b in control (A, C) and *Wt1^−/flox^; Cre-ER^TM^* (B, D) testes at 1 week after Tamoxifen treatment was assessed by Immunofluorescence. Compared to control testes, the expression of E-cadherin and Par6b in *Wt1*-deficient testes was disorganized. (E) Real time PCR results showed that the mRNA level of *Par6b* and *Wnt4* was significantly reduced in *Wt1^−/flox^; Cre-ER^TM^* testes at 1 week after Tamoxifen treatment, mRNA level of *E-cadherin* and *Wnt11* was also reduced, but was not statistically significant. *p<0.05.
(TIF)

Figure S6 The histology of testes biopsy from human NOA patients. (A) The testes from NOA patient with WT1 mutation. (B) The testes from NOA patient without WT1 mutation. Asterisk indicated the seminiferous tubules and black arrows indicated the Sertoli cells.
(TIF)

Figure S7 Immunofluorescence of BTB components. The expression of tight junction protein Claudin11 (A, B), adhesion junction proteins N-cadherin (C, D) and β-catenin (E, F), gap junction protein CX43 (G, H) in control (A, C, E, G) and *Wt1^−/flox^; Cre-ER^TM^* (B, D, F, H) testes at 1 week after Tamoxifen induction was assessed by immunofluorescence. All of these proteins were detected at the peripheral region of seminiferous tubules where tight junctions are formed in control testes. In contrast, the expression of these proteins was significantly reduced in *Wt1^−/flox^; Cre-ER^TM^* testes.
(TIF)

Figure S8 The integrity of BTB is damaged in *Wt1^−/flox^; Cre-ER^TM^* testes after Tamoxifen induction. The integrity of BTB in *Wt1^−/flox^; Cre-ER^TM^* at 1 week after Tamoxifen induction was assessed by biotin tracer injection. In *Wt1*-deficient testes (B, D), about 30% of the tubules were biotin-positive (green, white asterisks), whereas, no biotin-positive tubules were observed in control testes (A, C). E. Differences between control and *Wt1*-deficient testes were statistically significant, *p<0.05.
(TIF)

Figure S9 The expression of Sertoli cells specific genes is not changed in *Wt1*-deficient Sertoli cells. The expression of Sox9 (A, B), Gata4 (C, D), and Dmrt1 (E, F) in control (A, C, E) and Tamoxifen treated *Wt1^−/flox^; Cre-ER^TM^* Sertoli cells (B, D, F) was examined by immunofluorescence. No significant difference was noted between control and *Wt1*-deficient Sertoli cells. (G) The real time PCR results showed that the mRNA level of *Dmrt1, Gata1, Gata4, Nr5a1, Sox9*, and *AR* was not changed in *Wt1*-deficient Sertoli cells.
(TIF)

Figure S10 The expression of EMT-related genes is not changed in *Wt1*-deficient SCs.
(TIF)

Figure S11 The WT1 R362Q and K386R mutations do not affect the nuclear localization of WT1 protein. HepG2 cells were transfected with control adenovirus (A–D), *Wt1* expressing adenovirus (E–H), *Wt1^K386R^* expressing adenovirus (I–L), or *Wt1^R362Q^* expressing adenovirus (M–P). Adenovirus transfected cells were GFP positive (A, E, I, M, green). The expression of exogenous Wt1 was examined by immunofluorescence (red), and the WT1 (G), Wt1^K386R^ (K), and Wt1^R362Q^ (O) proteins were all detected in the nucleus.
(TIF)

Table S1 Summary of depth of sequencing of WT1 coding exons across 727 samples. The average sequencing depth of each coding exon, the fraction of coding bases covered at least 1× and with sufficient coverage to variant call (≥8× and consensus quality ≥20) are averaged for case group and control group respectively.
(DOC)

Table S2 Novel mutations of WT1 gene identified in the patients with azoospermia and bioinformatics prediction of the functional impact of these novel mutations. A mutation is regarded as deleterious if the SIFT<0.05, or PolyPhen>0.85, or PhastCons>0.95, or GERP>4.
(DOC)

Table S3 List of selected differentially expressed genes in *Wt1*-deficient Sertoli cells. Control and *Wt1^−/flox^; Cre-ER^TM^* Sertoli cells were cultured in vitro and treated with Tamoxifen for 3 days before total RNA was isolated for RNA-Seq analysis.
(DOC)

Table S4 Primers used for real time PCR.
(DOC)

Table S5 Primers used for PCR and Sanger sequencing confirmation of the WT1 mutations identified by high-throughput sequencing.
(DOC)
