# Supplementary figures and images for: The Wilms Tumor Gene, Wt1, Is Critical for Mouse Spermatogenesis via Regulation of Sertoli Cell Polarity and Is Associated with Non-Obstructive Azoospermia in Humans
Source: PLoS Genet. 2013 Aug 1;9(8):e1003645. doi: 10.1371/journal.pgen.1003645 (PMC3731222; doi:10.1371/journal.pgen.1003645)

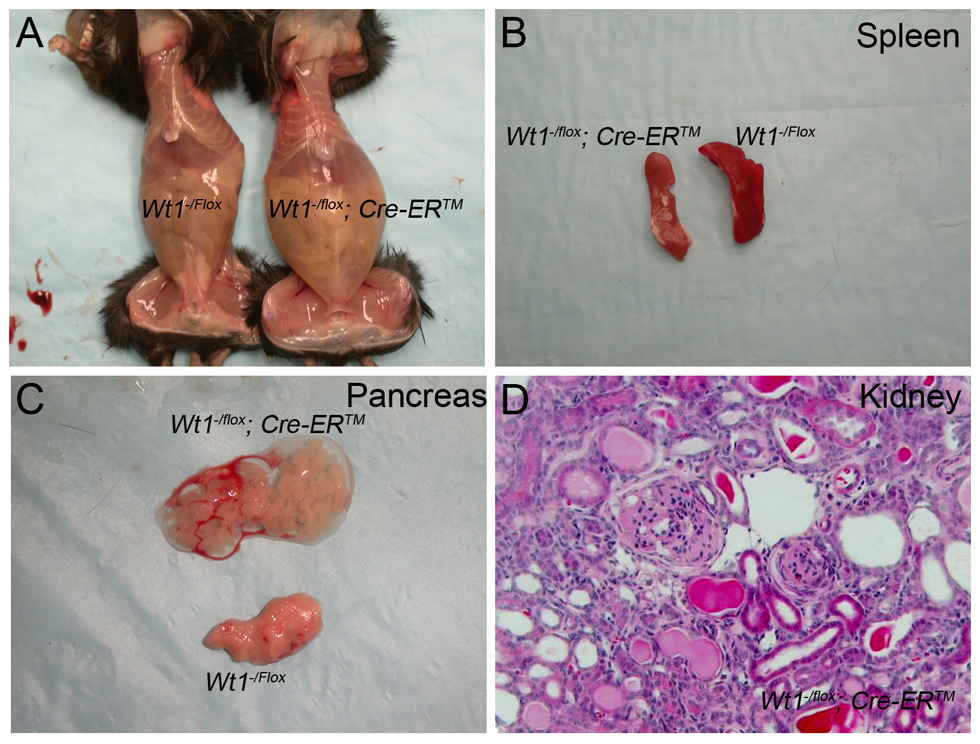

Supplement: Figure S1 — Inactivation of Wt1 results in multiple organ defects. (A) 3 weeks after Tamoxifen treatment, ascitic fluid was noted in Wt1-deficient mice (right). (B) Images of atropic spleen (left) in Wt1-deficient mice compared with control spleen (right). (C) Abnormal pancreas (upper) from Wt1-deficient mice compared to pancreas from control mice (lower). (D) Severe glomerulosclerosis in Wt1-deficient mice. (TIF) [file pgen.1003645.s001.tif]

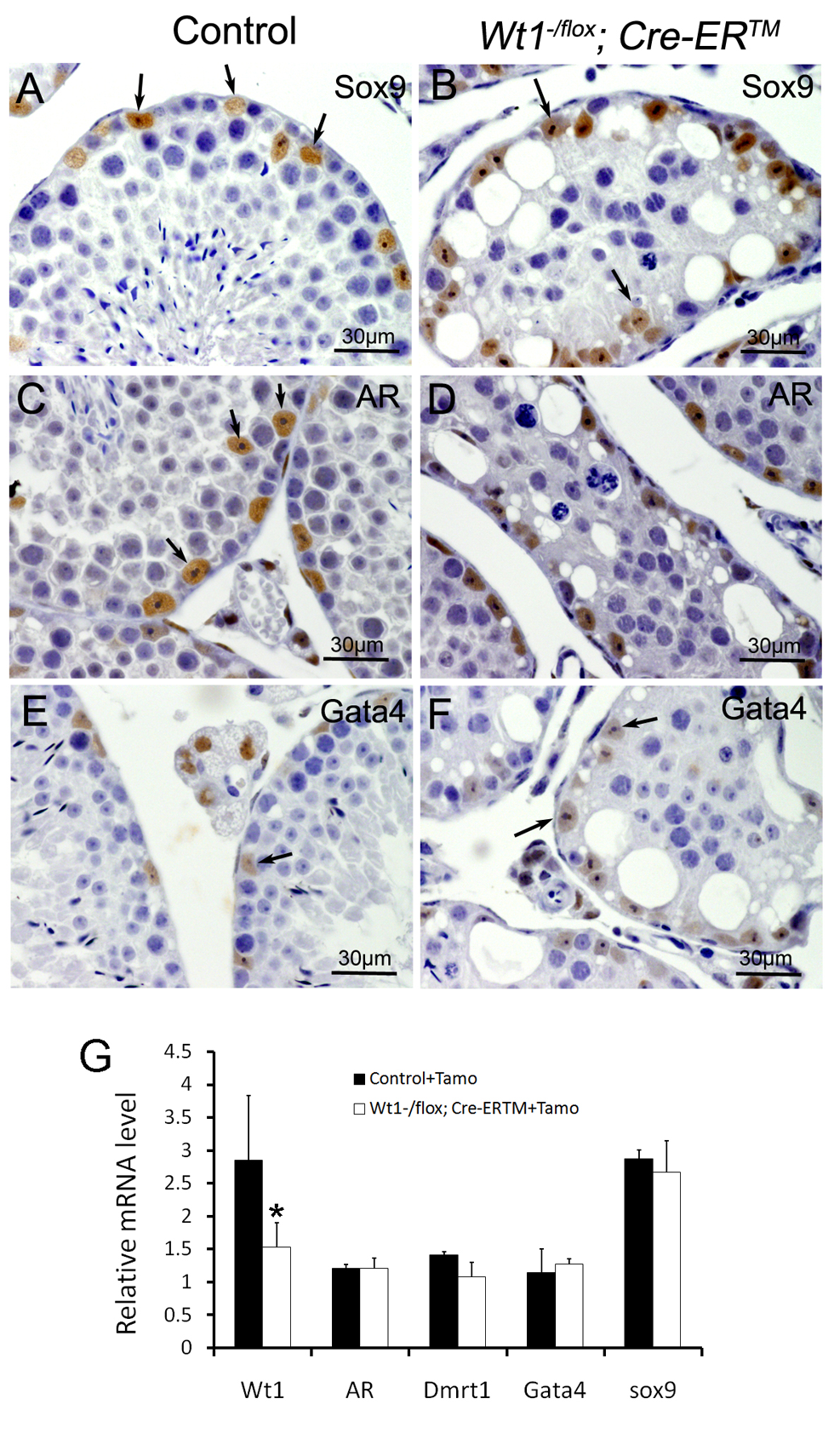

Supplement: Figure S2 — The expression of Sertoli cells-specific genes is not changed in Wt1-deficient testes. The expression of Sox9 (A,B), AR (C, D), and Gata4 (E, F) in Sertoli cells of control (A, C, E, arrows) and Wt1−/flox; Cre-ERTM (B, D, F, arrows) testes at 3 weeks after Tamoxifen treatment was analyzed by immunohistochemistry, and no obvious difference was observed between control and Wt1-deficient mice. (G) The mRNA level of AR, Dmrt1, Gata4, and Sox9 in control and Wt1−/flox; Cre-ERTM testes at 1 week after Tamoxifen treatment was analyzed by real time PCR. (TIF) [file pgen.1003645.s002.tif]

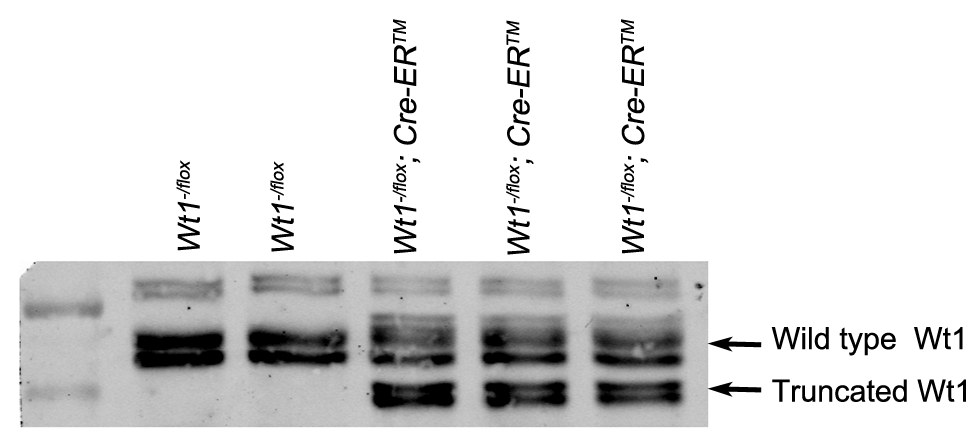

Supplement: Figure S3 — Western blot results of Wt1 expression in control and Wt1−/flox; Cre-ERTM testes at 1 week after Tamoxifen treatment, showing expression of the truncated Wt1 protein following deletion of exon 8 and 9. (TIF) [file pgen.1003645.s003.tif]

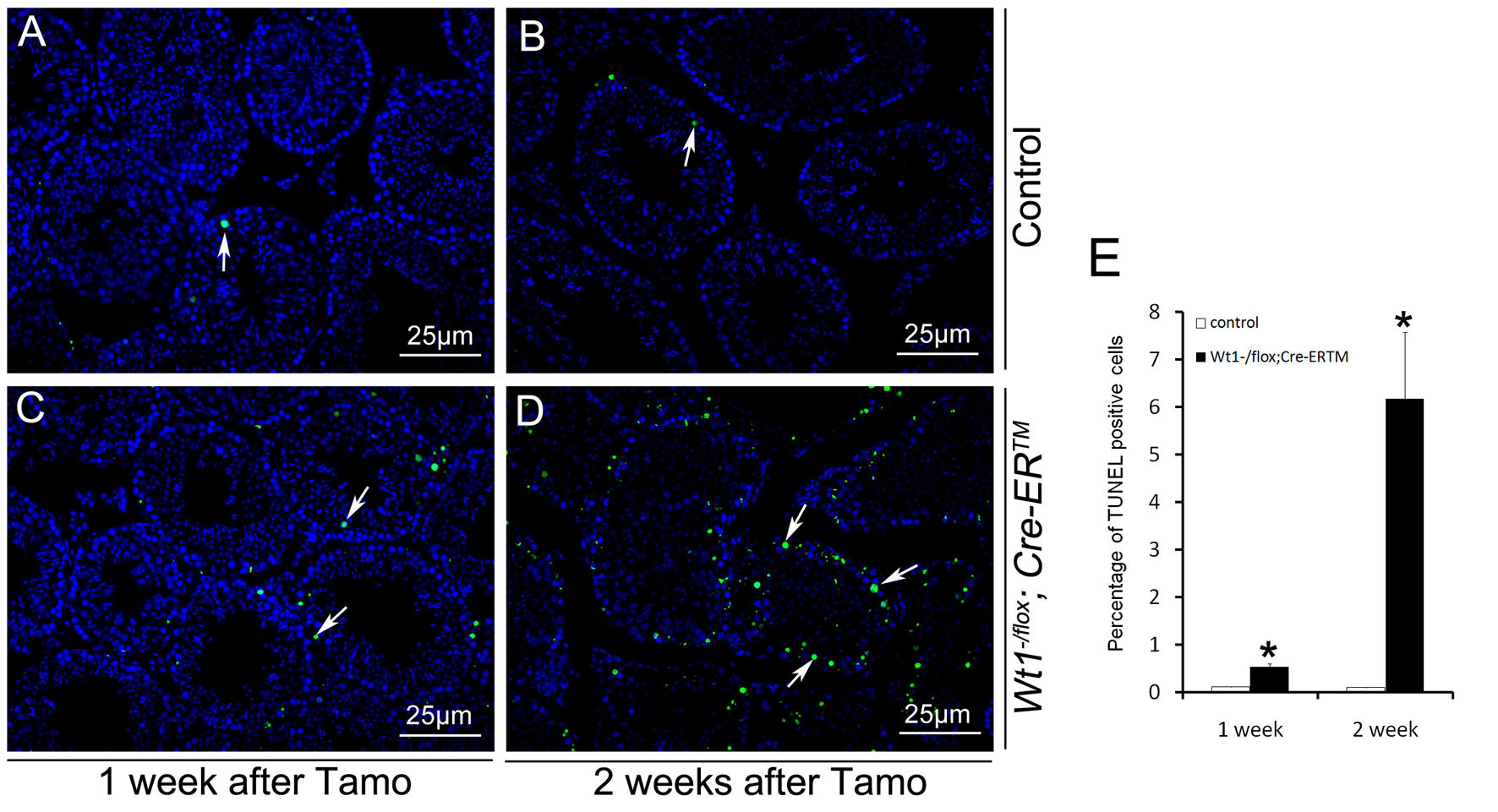

Supplement: Figure S4 — Apoptotic cells are detected in Wt1−/flox; Cre- ERTM testis after Tamoxifen treatment. Very few TUNEL-positive cells (green, white arrows) were observed in control testes at 1 (A) and 2 (B) weeks after Tamoxifen induction. TUNEL positive cells (green, white arrows) were observed in Wt1−/flox; Cre- ERTM testis at 1 week after Tamoxifen treatment (C), and the number of apoptotic cells (green, white arrows) was dramatically increased 2 weeks after Tamoxifen treatment (D). (E) The results of statistical analysis showed that the difference between control and Wt1-deficient testes was significant. * p<0.05. (TIF) [file pgen.1003645.s004.tif]

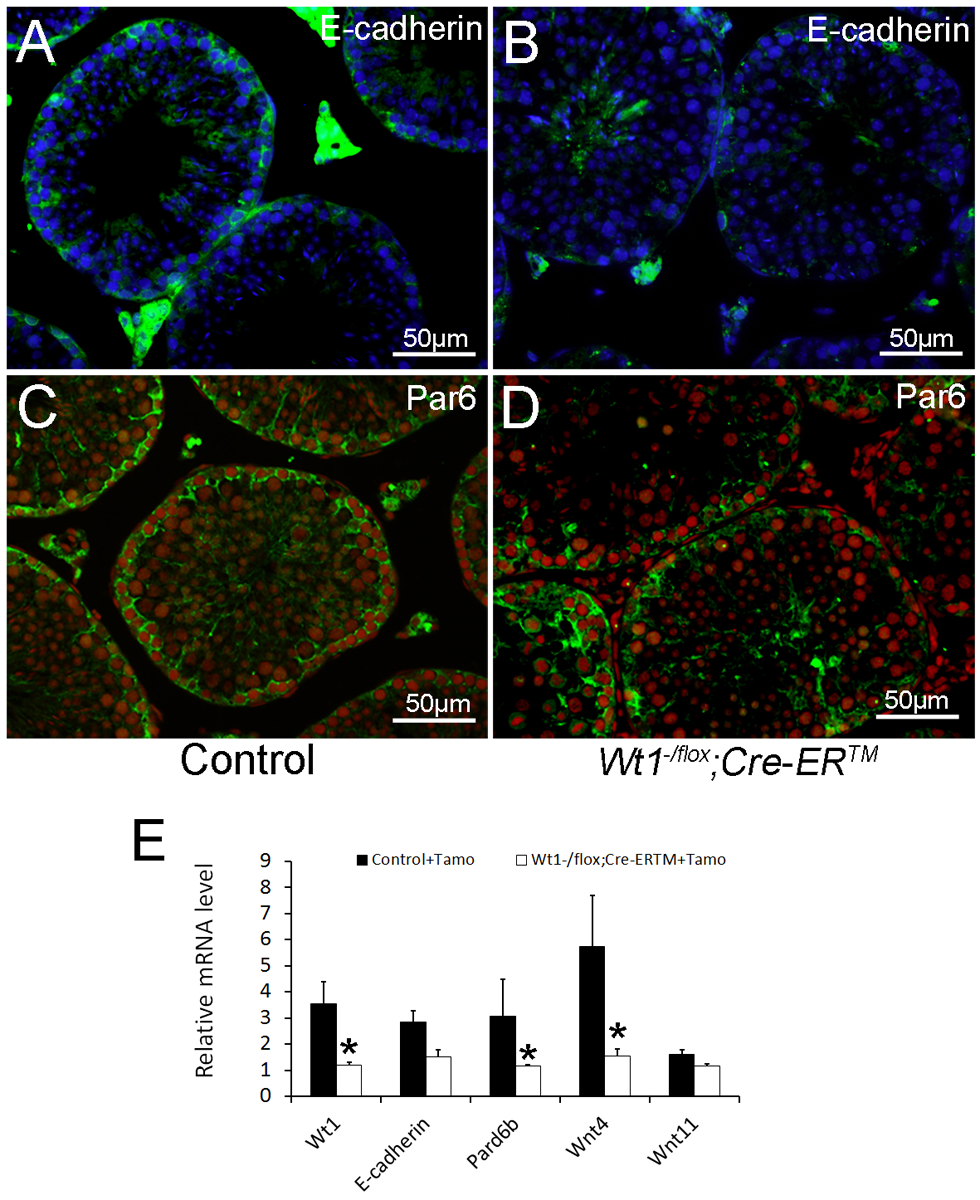

Supplement: Figure S5 — Immunofluorescence of E-cadherin and Par6b and RT-PCR results. The expression of E-cadherin and Par6b in control (A, C) and Wt1−/flox; Cre-ERTM (B, D) testes at 1 week after Tamoxifen treatment was assessed by Immunofluorescence. Compared to control testes, the expression of E-cadherin and Par6b in Wt1-deficient testes was disorganized. (E) Real time PCR results showed that the mRNA level of Par6b and Wnt4 was significantly reduced in Wt1−/flox; Cre-ERTM testes at 1 week after Tamoxifen treatment, mRNA level of E-cadherin and Wnt11 was also reduced, but was not statistically significant. *p<0.05. (TIF) [file pgen.1003645.s005.tif]

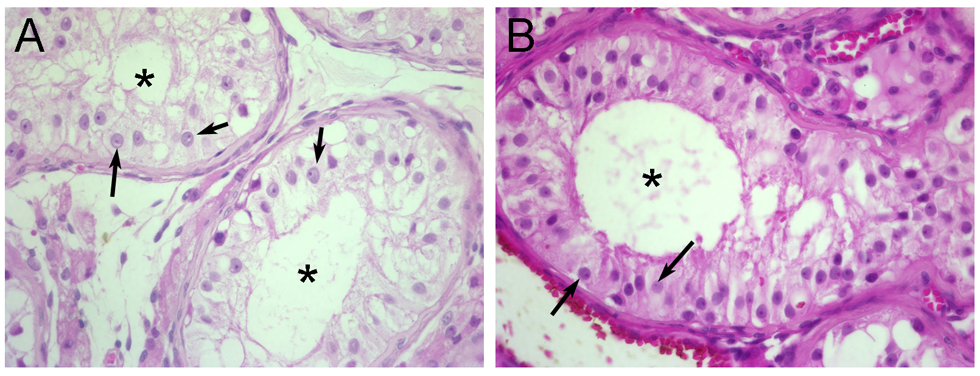

Supplement: Figure S6 — The histology of testes biopsy from human NOA patients. (A) The testes from NOA patient with WT1 mutation. (B) The testes from NOA patient without WT1 mutation. Asterisk indicated the seminiferous tubules and black arrows indicated the Sertoli cells. (TIF) [file pgen.1003645.s006.tif]

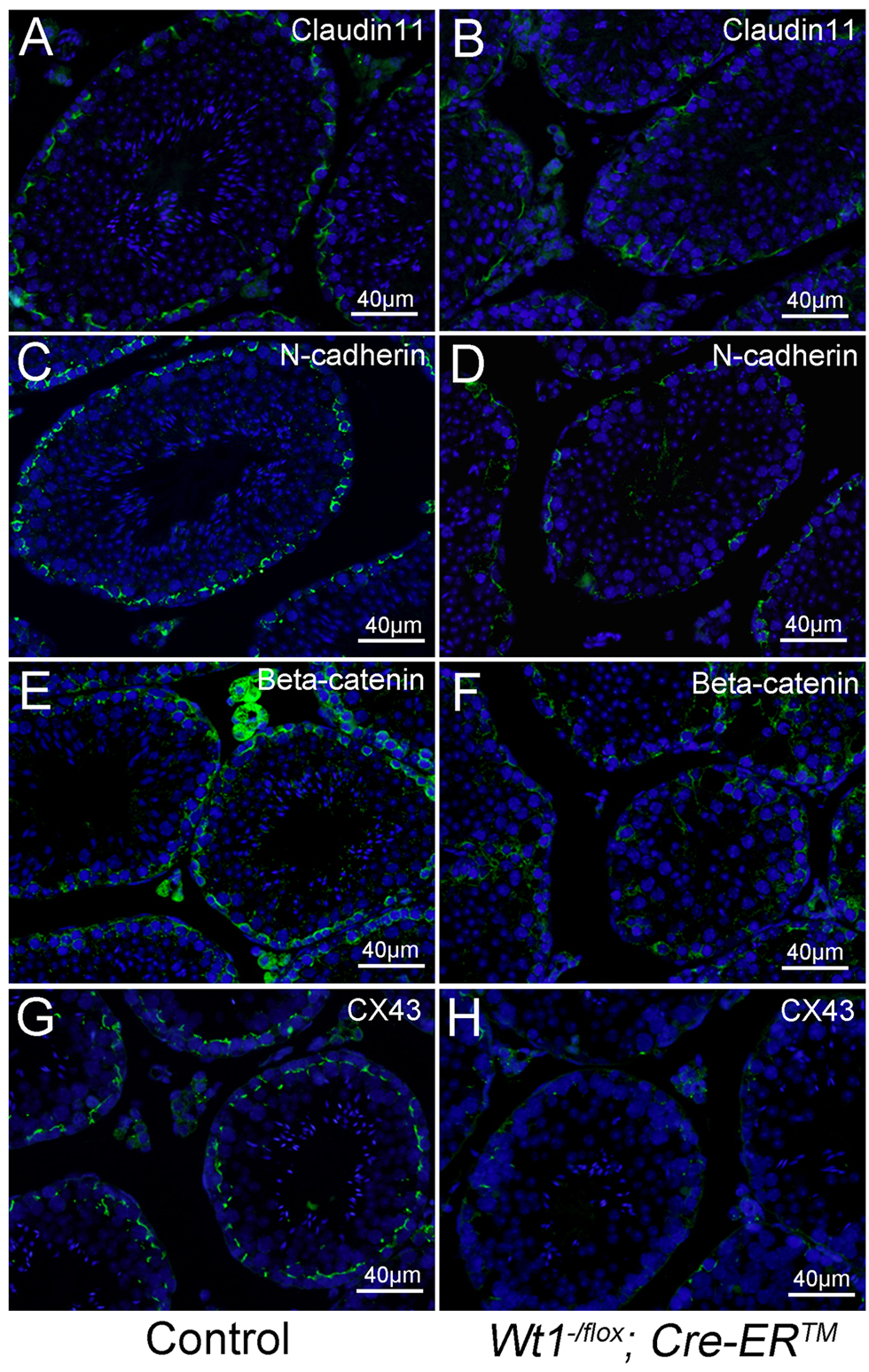

Supplement: Figure S7 — Immunofluorescence of BTB components. The expression of tight junction protein Claudin11 (A, B), adhesion junction proteins N-cadherin (C, D) and β-catenin (E, F), gap junction protein CX43 (G, H) in control (A, C, E, G) and Wt1−/flox; Cre-ERTM (B, D, F, H) testes at 1 week after Tamoxifen induction was assessed by immunofluorescence. All of these proteins were detected at the peripheral region of seminiferous tubules where tight junctions are formed in control testes. In contrast, the expression of these proteins was significantly reduced in Wt1−/flox; Cre-ERTM testes. (TIF) [file pgen.1003645.s007.tif]

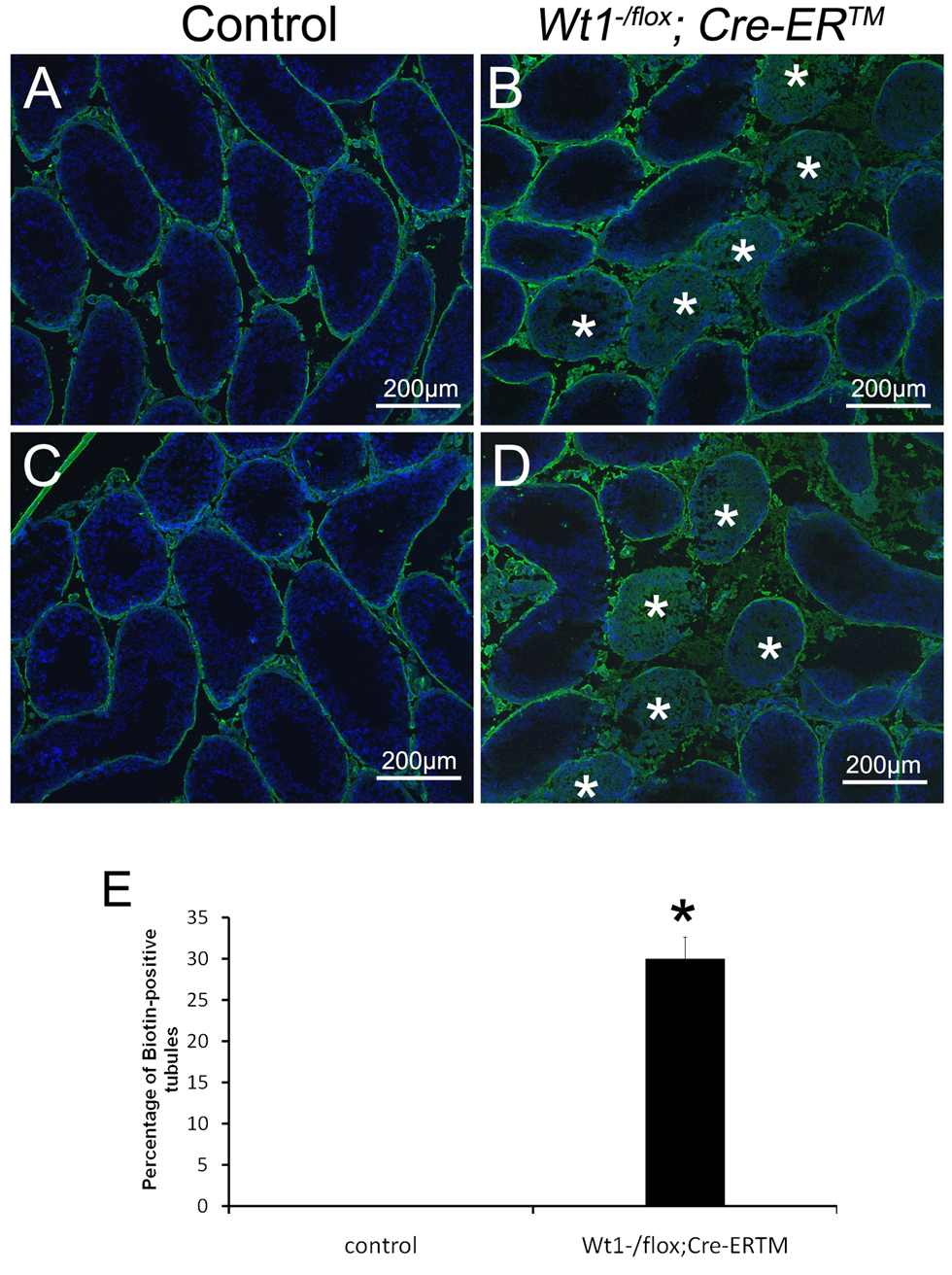

Supplement: Figure S8 — The integrity of BTB is damaged in Wt1−/flox; Cre-ERTM testes after Tamoxifen induction. The integrity of BTB in Wt1−/flox; Cre-ERTM at 1 week after Tamoxifen induction was assessed by biotin tracer injection. In Wt1-deficient testes (B, D), about 30% of the tubules were biotin-positive (green, white asterisks), whereas, no biotin-positive tubules were observed in control testes (A, C). E. Differences between control and Wt1-deficient testes were statistically significant, *p<0.05. (TIF) [file pgen.1003645.s008.tif]

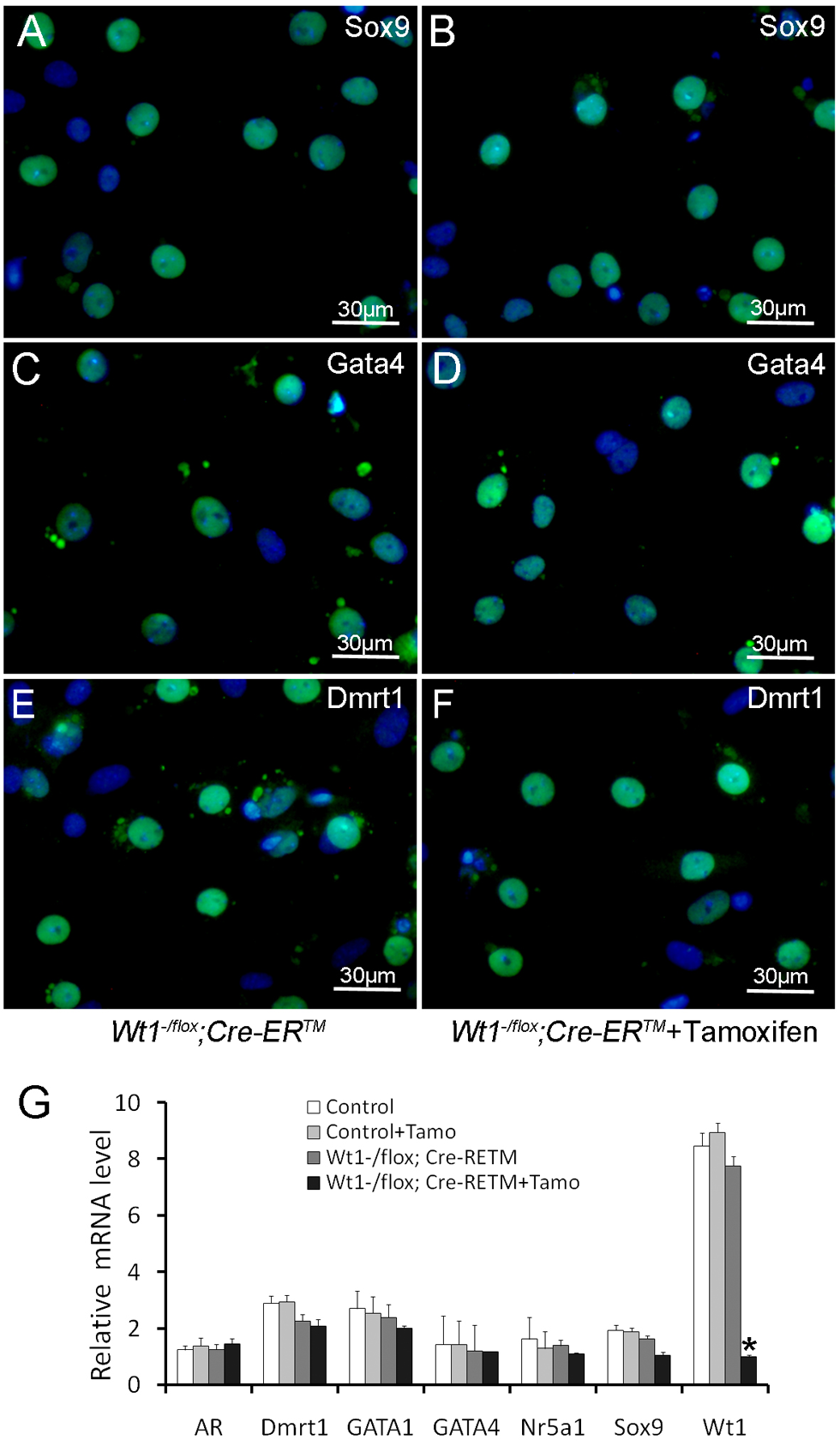

Supplement: Figure S9 — The expression of Sertoli cells specific genes is not changed in Wt1-deficient Sertoli cells. The expression of Sox9 (A, B), Gata4 (C, D), and Dmrt1 (E, F) in control (A, C, E) and Tamoxifen treated Wt1−/flox; Cre-ERTM Sertoli cells (B, D, F) was examined by immunofluorescence. No significant difference was noted between control and Wt1-deficient Sertoli cells. (G) The real time PCR results showed that the mRNA level of Dmrt1, Gata1, Gata4, Nr5a1, Sox9, and AR was not changed in Wt1-deficient Sertoli cells. (TIF) [file pgen.1003645.s009.tif]

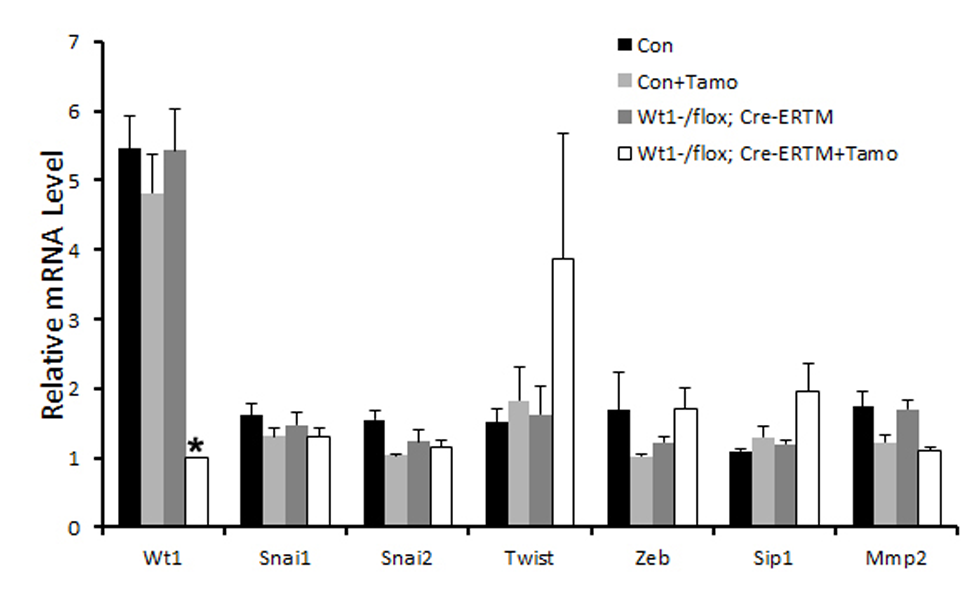

Supplement: Figure S10 — The expression of EMT-related genes is not changed in Wt1-deficient SCs. (TIF) [file pgen.1003645.s010.tif]

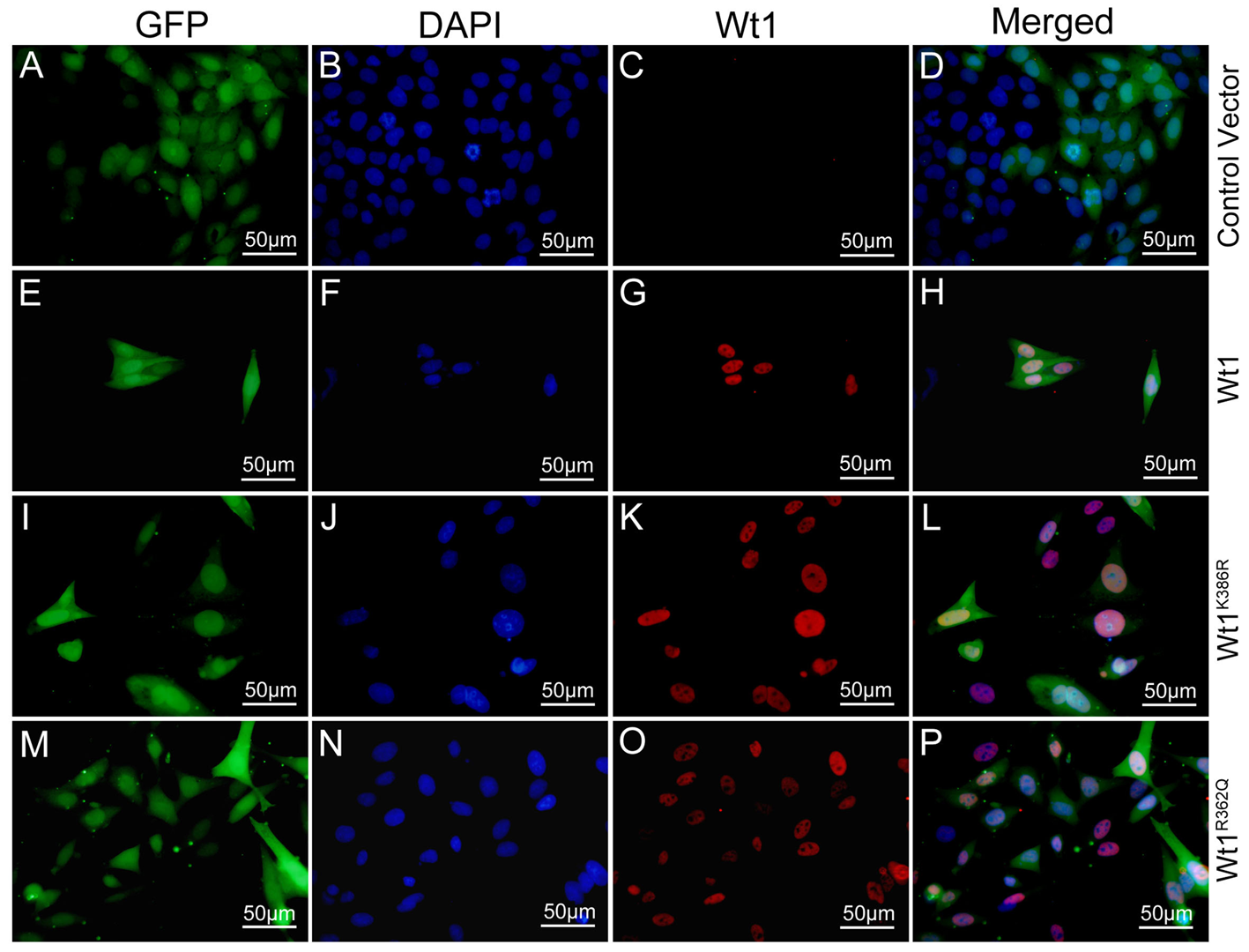

Supplement: Figure S11 — The WT1 R362Q and K386R mutations do not affect the nuclear localization of WT1 protein. HepG2 cells were transfected with control adenovirus (A–D), Wt1 expressing adenovirus (E–H), Wt1K386R expressing adenovirus (I–L), or Wt1R362Q expressing adenovirus (M–P). Adenovirus transfected cells were GFP positive (A, E, I, M, green). The expression of exogenous Wt1 was examined by immunofluorescence (red), and the WT1 (G), Wt1K386R (K), and Wt1R362Q (O) proteins were all detected in the nucleus. (TIF) [file pgen.1003645.s011.tif]
