# Supplementary material for: The Wilms Tumor Gene, Wt1, Is Critical for Mouse Spermatogenesis via Regulation of Sertoli Cell Polarity and Is Associated with Non-Obstructive Azoospermia in Humans
Source: PLoS Genet. 2013 Aug 1;9(8):e1003645. doi: 10.1371/journal.pgen.1003645 (PMC3731222; doi:10.1371/journal.pgen.1003645)
Supplement: Table S1 — Summary of depth of sequencing of WT1 coding exons across 727 samples. The average sequencing depth of each coding exon, the fraction of coding bases covered at least 1× and with sufficient coverage to variant call (≥8× and consensus quality ≥20) are averaged for case group and control group respectively. (DOC) [file pgen.1003645.s012.doc]

Supplementary Table 1.

|  |  |  | NOA Cases (n=529) | | | Controls (n=198) | | |
| --- | --- | --- | --- | --- | --- | --- | --- | --- |
| # Exon | Size (bp) | % GC | Ave. Depth | Covered ≥ 1× (%) | Sequence called (%) | Ave. Depth | Covered ≥ 1× (%) | Sequence called (%) |
| 1 | 646 | 72.76 | 10.13 | 90.50 | 48.86 | 9.59 | 90.18 | 46.40 |
| 2 | 123 | 64.23 | 46.46 | 100.00 | 100.00 | 46.10 | 100.00 | 100.00 |
| 3 | 103 | 67.96 | 16.03 | 100.00 | 93.41 | 15.51 | 100.00 | 89.80 |
| 4 | 78 | 42.31 | 97.52 | 100.00 | 100.00 | 96.50 | 100.00 | 100.00 |
| 5 | 51 | 54.90 | 76.24 | 100.00 | 100.00 | 76.68 | 100.00 | 100.00 |
| 6 | 97 | 55.67 | 68.13 | 100.00 | 100.00 | 67.22 | 100.00 | 100.00 |
| 7 | 151 | 52.98 | 73.34 | 100.00 | 100.00 | 72.92 | 100.00 | 100.00 |
| 8 | 90 | 45.56 | 90.02 | 100.00 | 100.00 | 87.73 | 100.00 | 99.99 |
| 9 | 84 | 51.19 | 62.15 | 100.00 | 100.00 | 61.43 | 100.00 | 100.00 |
| 10 | 122 | 49.18 | 80.35 | 100.00 | 100.00 | 80.69 | 100.00 | 99.99 |
| **Total** | **1545** | **62.01** | **42.85** | **96.01** | **78.18** | **42.29** | **95.89** | **76.91** |
