# Supplementary material for: The Wilms Tumor Gene, Wt1, Is Critical for Mouse Spermatogenesis via Regulation of Sertoli Cell Polarity and Is Associated with Non-Obstructive Azoospermia in Humans
Source: PLoS Genet. 2013 Aug 1;9(8):e1003645. doi: 10.1371/journal.pgen.1003645 (PMC3731222; doi:10.1371/journal.pgen.1003645)
Supplement: Table S2 — Novel mutations of WT1 gene identified in the patients with azoospermia and bioinformatics prediction of the functional impact of these novel mutations. A mutation is regarded as deleterious if the SIFT<0.05, or PolyPhen>0.85, or PhastCons>0.95, or GERP>4. (DOC) [file pgen.1003645.s013.doc]

Supplementary Table 2.

| Patient No. | Exon | Position (hg19) | Codon change | Amino acid change | SIFT | PolyPhen2 | PhastCons | GERP |
| --- | --- | --- | --- | --- | --- | --- | --- | --- |
| W579 | 3 | 32449530 | GCT> CCT | Ala214Pro | NA | 0.938 | 1.0 | 5.060 |
| W378 | 4 | 32439153 | AAT>AGT | Asn239Ser | NA | 0.866 | 1.0 | 5.610 |
| W721 | 6 | 32421579 | GGG>GCG | Gly270Ala | NA | 0.182 | 0.998 | 5.630 |
| W204 | 6 | 32421503 | AGA>AGT | Arg295Ser | 0 | 1 | 0.999 | -0.097 |
| W643 | 8 | 32414262 | CGA>CAA | Arg362Gln | NA | 0.408 | 1.0 | 5.680 |
| W606 | 9 | 32413589 | AAA>AGA | Lys386Arg | NA | 0.1 | 1.0 | 5.690 |
