# Supplementary material for: The Wilms Tumor Gene, Wt1, Is Critical for Mouse Spermatogenesis via Regulation of Sertoli Cell Polarity and Is Associated with Non-Obstructive Azoospermia in Humans
Source: PLoS Genet. 2013 Aug 1;9(8):e1003645. doi: 10.1371/journal.pgen.1003645 (PMC3731222; doi:10.1371/journal.pgen.1003645)
Supplement: Table S3 — List of selected differentially expressed genes in Wt1-deficient Sertoli cells. Control and Wt1−/flox; Cre-ERTM Sertoli cells were cultured in vitro and treated with Tamoxifen for 3 days before total RNA was isolated for RNA-Seq analysis. (DOC) [file pgen.1003645.s014.doc]

Supplementary Table 3

| Pathway term | Symbol | log2 Ratio(KOT/KO)  (P-value﹤0.05) | Accession no. |
| --- | --- | --- | --- |
| Pathways in cancer | Col10a1  Wnt10a  Wnt7b  Dpp4  Wnt7b  Celsr1  Rspo1  Dhh  Frzb  Lama3  Cited1  Fgf9  Mdfi  Eln  Cdh1  Egf  Hhex  Wnt11  Bmp4  Cdkn2a  Fap  Mmp2  Itga7  Col14a1  Pparg  Ctnna2  Dvl3  Wnt5a  Kitl | -7.942514505  -7.129283017  -7.129283017  -7.129283017  -7.129283017  -6.459431619  -4.494598473  -4.063509943  -3.323679293  -3.174497731  -3.012751498  -2.651457913  -2.645504043  -2.630947368  -2.541257277  -2.491248066  -2.310998535  -2.276654015  -2.259548517  -2.152464883  -2.089885504  -2.030982225  -1.947463252  7.948367232  2.97466871  2.871013559  2.258734268  2.121287983  1.829108249 | NM_009925  NM_009518  NM_001163633  NM_010074  NM_001163633  NM_009886  NM_138683  NM_007857  NM_011356  NM_010680  NM_007709  NM_013518  NM_010783  NM_007925  NM_009864  NM_010113  NM_008245  NM_009519  NM_007554  NM_001040654  NM_007986  NM_008610  NM_008398  NM_181277  NM_011146  NM_001109764  NM_007889  NM_009524  NM_013598 |
| ECM-receptor interaction | Col10a1  Dpp4  Lama3  Eln  Fap  Itga7  Col14a1 | -7.942514505  -7.129283017  -3.174497731  -2.630947368  -2.089885504  -1.947463252  7.948367232 | NM_009925  NM_010074  NM_010680  NM_007925  NM_007986  NM_008398  NM_181277 |
| Axon guidance | Sema6a  Ephb1  Lgi4  Dhh  Sema7a  Serinc5  Sema6c  Rgnef  Mmp2  Sema5b  Mal  Plp1  Isl2  Scd4  Ablim2  Cxcl12  Slit1  Wnt5a | -7.592457037  -6.459431619  -6.459431619  -4.063509943  -3.181018504  -2.787912084  -2.578125071  -2.064130337  -2.030982225  9.920352855  8.22881869  8.044394119  5.727920455  4.659463931  2.300844494  2.192268805  2.140481224  2.121287983 | NM_018744  NM_001168296  NM_144556  NM_007857  NM_001177700  NM_172588  NM_011351  NM_012026  NM_008610  NM_013661  NM_001171187  NM_011123  NM_027397  NM_183216  NM_009524  NM_013655  NM_015748  NM_011352 |
| p53 signaling pathway | Igfbp3  Ccnb1  Brca2  Ccne1 | -4.135324002  -1.836181521  -1.784677702  -1.732038913 | NM_008343  NM_172301  NM_009765  NM_007633 |
| Focal adhesion | Itgad  Itgam  Actn2  Shcbp1  Actn3  Itga7 | -7.592457037  -6.714245518  -6.714245518  -2.977279923  -2.029191713  -1.947463252 | NM_001177632  NM_001082960  NM_033268  NM_011369  NM_013456  NM_008398 |
| ErbB signaling pathway | Shcbp1  Egf  Lif  Areg  Nckap1  Arid5b  Igf2 | -2.977279923  -2.491248066  -1.995895602  6.459431619  1.769387072  1.766340196  1.722066718 | NM_011369  NM_010113  NM_008501  NM_009704  NM_016965  NM_023598  NM_001122737 |
| Endocytosis | Ston1  Pacsin1  Fcgr2b  C3  Egf  Sorl1  Pard6b  Cdh13  A230073K1  Ramp2 | -7.451211112  -6.714245518  9.997179481  -2.765295315  -2.491248066  -2.19520441  -1.73065182  9.997179481  6.942514505  2.183599938 | NM_029858  NM_011861  NM_010187  NM_009778  NM_010113  NM_011436  NM_021409  NM_019707  NR_033229  NM_019444 |
| PPAR signaling pathway | Olr1  Racgap1  Pparg  Pltp  Fabp7 | -3.350296608  -2.183703043  2.97466871  2.229623011  2.214301834 | NM_138648  NM_012025  NM_011146  NM_011125  NM_021272 |
| Hedgehog signaling pathway | Dpp4  Wnt10a  Wnt7b  Rspo1  Dhh  Frzb  Cited1  Fgf9  Mdfi  Hhex  Wnt11  Gas1  Lrp4  Gli3  Sfrp2  Dvl3  Wnt5a | -7.129283017  -7.129283017  -7.129283017  -4.494598473  -4.063509943  -3.323679293  -3.012751498  -2.651457913  -2.645504043  -2.310998535  -2.276654015  -2.018235571  -1.882643049  -1.817939673  3.241638914  2.258734268  2.121287983 | NM_010074  NM_009518  NM_001163633  NM_138683  NM_007857  NM_011356  NM_007709  NM_013518  NM_010783  NM_008245  NM_009519  NM_008086  NM_172668  NM_008130  NM_009144  NM_007889  NM_009524 |
| TGF-beta signaling pathway | Mecom  Cited1  Prdm16  Bmp4 | -7.129283017  -3.012751498  -2.795641501  -2.259548517 | NM_007963  NM_007709  NM_001177995  NM_007554 |
| Cell cycle | Mecom  Sycp3  Dhh  Aspm  Spag5  Cdc25c  Cenpf  Plk1  Nuf2  Dlgap5  Bub1b  Racgap1  Mis12  Cdkn2a  Gas1  Bcat1  Cdkn3  Prkcq  Kif2c | -7.129283017  -6.942514505  -4.063509943  -2.813231488  -2.750512126  -2.57357574  -2.46317402  -2.401587544  -2.394278939  -2.321928095  -2.269004775  -2.183703043  -2.160822794  -2.152464883  -2.018235571  -1.990510086  -1.946228744  8.30833903  -2.394278939 | NM_007963  NM_011517  NM_007857  NM_009791  NM_017407  NM_009860  NM_001081363  NM_025993  NM_023284  NM_001145949  NM_009773  NM_012025  NM_134471  NM_001040654  NM_008086  NM_001024468  NM_028222  NM_008859  NM_011121 |
