# Supplementary material for: The Wilms Tumor Gene, Wt1, Is Critical for Mouse Spermatogenesis via Regulation of Sertoli Cell Polarity and Is Associated with Non-Obstructive Azoospermia in Humans
Source: PLoS Genet. 2013 Aug 1;9(8):e1003645. doi: 10.1371/journal.pgen.1003645 (PMC3731222; doi:10.1371/journal.pgen.1003645)
Supplement: Table S4 — Primers used for real time PCR. (DOC) [file pgen.1003645.s015.doc]

Supplementary Table 4

| Gene Name | Forward Primer | Reverse Primer |
| --- | --- | --- |
| Wt1  Gata4  Sf1  Wnt4  Cx43  Claudin11  GAPDH  Pard6b  AR  GATA1  Dmrt1  Nr5a1  Sox9 | 5’CCAGTGTAAAACTTGTCAGCGAAA 3’  5’TCCAGTGCTGTCTGCTCTGAAG3’  5’CTGTGCGTGCTGATCGAATG3’  5’CCGGGCACTCATGAATCTTC3’  5’CCACTGAGCCCATCCAAAGA3’  5’CATGGTAGCCACTTGCCTTCA3’  5’TTGTCTCCTGCGACTTCAACA3’  5’ CTCCTGCTACGACCAGTGG3’  5’ CTGGGAAGGGTCTACCCAC3’  5’ TGGGGACCTCAGAACCCTTG3’  5’ GGAGTCTCCCAGCACCTTACG3’  5’ CCTCGATGTGAAATTCCTGAACA3’  5’ GCATCTGCACAACGCGG3’ | 5’ATGAGTCCTGGTGTGGGTCTTC3’  5’CTGGCCTGCGATGTCTGAGT3’  5’GCCCGAATCTGTGCTTTCTTC3’  5’CACCCGCATGTGTGTCAAG3’  5’TGGTGAGGAGCAGCCATTG3’  5’CCAGTCATTGGTGGACGTTGT3’  5’ACCAGGAAATGAGCTTGACAAAG3’  5’ ACACCTGGCGGAAATCTTGG3’  5’ GGTGCTATGTTAGCGGCCTC3’  5’ GGCTGCATTTGGGGAAGTG3’  5’ TCTGCCACTGGTTTCCAGTCT3’  5’ TCCTGGGCGTCCTTTACG3’  5’ AGCCTCCAGAGCTTGCCC3’ |
