# Supplementary material for: The Wilms Tumor Gene, Wt1, Is Critical for Mouse Spermatogenesis via Regulation of Sertoli Cell Polarity and Is Associated with Non-Obstructive Azoospermia in Humans
Source: PLoS Genet. 2013 Aug 1;9(8):e1003645. doi: 10.1371/journal.pgen.1003645 (PMC3731222; doi:10.1371/journal.pgen.1003645)
Supplement: Table S5 — Primers used for PCR and Sanger sequencing confirmation of the WT1 mutations identified by high-throughput sequencing. (DOC) [file pgen.1003645.s016.doc]

Supplementary Table 5

| Exon | Forward Primer | Reverse Primer | | Product size | | |
| --- | --- | --- | --- | --- | --- | --- |
| 3  4  6  8  9 | 5’ TCTCGTGCCTCCAAGACCC 3’  5’ GGGAAGGAGGAAAGCGTTCTAATGT 3’  5’ AGCCCGCCCTGGAGAAGTC 3’  5’ AGCTGCCAGCAATGAGAAGTGA 3’  5’ AGCCACGCACTATTCCTTCTCTCAACT 3’ | | 5’ ACAGCGCAGCGACTGCTAG’  5’ TGTTGAGTTATCCAGCCTGTGTTGC 3’  5’ CGGTGCACGCCTACGAGAA 3’  5’ AGCTCCAGCGAAGTGCCTTAGG 3’  5’ TTGGGCCTGGGGGACTGGGG 3’ | | 400  486  618  241  395 |  |
